# Robust Unidirectional Airflow through Avian Lungs: New Insights from a Piecewise Linear Mathematical Model

**DOI:** 10.1371/journal.pcbi.1004637

**Published:** 2016-02-10

**Authors:** Emily P. Harvey, Alona Ben-Tal

**Affiliations:** Institute of Natural and Mathematical Sciences, Massey University Albany, Auckland, New Zealand; University of British Columbia, CANADA

## Abstract

Avian lungs are remarkably different from mammalian lungs in that air flows unidirectionally through rigid tubes in which gas exchange occurs. Experimental observations have been able to determine the pattern of gas flow in the respiratory system, but understanding how the flow pattern is generated and determining the factors contributing to the observed dynamics remains elusive. It has been hypothesized that the unidirectional flow is due to aerodynamic valving during inspiration and expiration, resulting from the anatomical structure and the fluid dynamics involved, however, theoretical studies to back up this hypothesis are lacking. We have constructed a novel mathematical model of the airflow in the avian respiratory system that can produce unidirectional flow which is robust to changes in model parameters, breathing frequency and breathing amplitude. The model consists of two piecewise linear ordinary differential equations with lumped parameters and discontinuous, flow-dependent resistances that mimic the experimental observations. Using dynamical systems techniques and numerical analysis, we show that unidirectional flow can be produced by either effective inspiratory or effective expiratory valving, but that both inspiratory and expiratory valving are required to produce the high efficiencies of flows observed in avian lungs. We further show that the efficacy of the inspiratory and expiratory valving depends on airsac compliances and airflow resistances that may not be located in the immediate area of the valving. Our model provides additional novel insights; for example, we show that physiologically realistic resistance values lead to efficiencies that are close to maximum, and that when the relative lumped compliances of the caudal and cranial airsacs vary, it affects the timing of the airflow across the gas exchange area. These and other insights obtained by our study significantly enhance our understanding of the operation of the avian respiratory system.

## Author Summary

Birds and mammals have similar metabolic demands and cardiovascular systems, but they have evolved drastically different respiratory systems. A key difference in birds is that gas exchange occurs in rigid tubes, through which air flows unidirectionally during both inspiration and expiration. How this unidirectional flow is generated, and the factors affecting it, are not well understood. It has been hypothesized that the unidirectional flow is due to aerodynamic valving resulting from the complex anatomical structure. To test this hypothesis we have constructed a novel mathematical model that, unlike previous models, produces unidirectional flow through the lungs consistently even when the amplitude and frequency of breathing change. We have investigated the model both analytically and computationally and shown the importance of aerodynamic valving for generating strong airflow through the lungs. Our model also predicts that the timing of airflow through the lungs depends on the relative compliances of the different airsacs that exist in birds. The lumped parameters approach we use means that this model is generally applicable across all birds.

## Introduction

The anatomical structure and airflow dynamics of the avian respiratory system are remarkably different to that of mammalian lungs [1]. The anatomical structure is complex, with multiple flexible airsacs that act like bellows to ventilate rigid tubes (*parabronchi*) in which gas exchange occurs, and a complicated branching structure that produces aerodynamic valving [1, 2]. The airflow through the parabronchi (lungs) is unidirectional; flowing from the *caudal* (back) group of airsacs to the *cranial* (front) group of airsacs during both inspiration and expiration. (More precisely, the flow is unidirectional through the *paleopulmonic*-parabronchi that lie between the caudal and cranial airsacs. Some birds also contain *neopulmonic*-parabronchi in which airflow is bidirectional, but it forms a small part of the gas exchange surface area—less than 30% [2]. In this paper we use the term *parabronchi* to refer to the paleopulmonic parabronchi unless otherwise indicated.)

Unlike in the mammalian respiratory system, the functions of ventilation and gas exchange have been uncoupled in the avian respiratory system; specifically, the flow of air through the system is caused by large flexible airsacs, whilst gas exchange occurs in narrow parabronchi which are rigid and firmly bound to the ribs [2]. The narrow, rigid structure of the parabronchi is thought to be related to the finding that birds have a thinner but mechanically stronger blood-gas barrier than equivalent mammals [3, 4]. Furthermore the structure of the parabronchi and blood capillaries allows for *cross-current* gas exchange. These features are thought to contribute to the increased gas exchange efficiency of birds compared to mammals, especially at high-altitude or in a hypoxic environment [3, 5–8].

The airflow pattern within the avian respiratory system is widely agreed upon. It has been determined by direct measurements of flow rates [9–11], as well as by experiments that used tracer gas, or CO_2_ and O_2_ measurements to indirectly determine the flow [10, 12–15]. An important factor leading to unidirectional flow is hypothesized to be the effective inspiratory and expiratory *aerodynamic valving* that results from the interaction between the complex anatomical structure, including airway branching and constrictions, and the fluid dynamics involved [1, 2]. The relative importance of the two valves has not been investigated. Additionally, the complex branching structure within the system affects the resistance to airflow of the different sections of the system. The effect of these resistances and the importance of their relative differences in generating the flow pattern is not known. Recently unidirectional airflow has been found in the lungs of some reptiles (specifically alligators [16, 17], crocodiles [18, 19], iguanas [20], and monitor lizards [21]). Comparing avian and reptile systems, which have very different levels of anatomical complexity in terms of the branching structures and the presence or absence of airsac separation, will provide important insights into the aerodynamic valving in both birds and reptiles.

Mathematical modelling of the avian respiratory system has focused mainly on the gas exchange in the parabronchi (for example, [6, 22–28]). These studies determined that gas exchange is cross-current and found gas exchange parameters for a range of avian species and experimental conditions [1, 2]. Existing mathematical models of the airflow through the avian system had limited success in producing unidirectional flow [29, 30]. Urushikubo et al. [30] used a three dimensional spatial model with simplified geometry for the pathways within the respiratory system, coupled with flexible airsacs. They found unidirectional flow through the parabronchi, but only for some parameter values. Additionally, the flow did not show any inspiratory or expiratory valving. Maina et al. [29] investigated aerodynamic inspiratory valving in ostriches by constructing a three dimensional anatomical model of the junction between the ventrobronchial branches and the main mesobronchus (i.e. the junction between the airways that lead air to the caudal airsacs and the airways that lead air from the cranial airsacs). Using computational fluid dynamics simulations they were only able to reproduce inspiratory valving if they included additional branches downstream (the secondary dorsobronchi branches), showing the importance of including the whole system when investigating aerodynamic valving.

Unidirectional flow exists in all birds, despite massive inter-species differences in anatomy, and across most experimental conditions—including when ventilating the respiratory system post-mortem [31]. Thus, a useful mathematical model of the airflow in the avian respiratory system must produce unidirectional flow through the parabronchi across a broad range of parameter values and frequencies. In this paper we present a new, relatively simple, mathematical model of avian respiration that reproduces the airflow pattern described above. The unidirectional flow in our model is robust to changes in frequency and model parameters, and has efficiencies, flow rates, and pressures that match experimental findings. Additionally, our model generates several novel insights on the role of inspiratory and expiratory valving and the importance of variations in the airflow resistances and airsac compliances within the system that are thought to occur during respiration and in response to stimuli including hypoxia (lack of oxygen) and hypercapnia (excess of carbon dioxide). We first describe the mathematical model and then the new insights it produced. The model development and mathematical analysis are described later in the Methods section.

## Results

### The model

**Fig 1:**
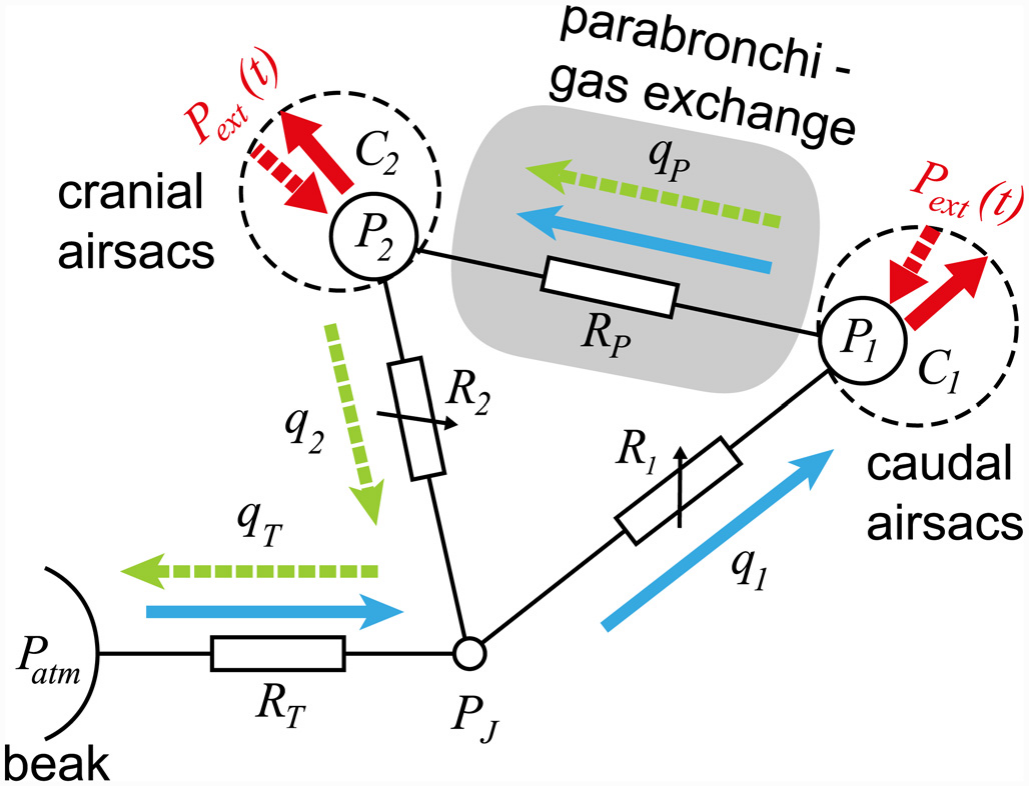
Schematic model of the avian respiratory system. The caudal and cranial airsacs have pressures *P*_1_ and *P*_2_ respectively, and compliances *C*_1_ and *C*_2_ respectively. The pressure outside both sets of airsacs, *P*_*ext*_(*t*), varies periodically due to the respiratory muscles, which causes the airsacs to inflate and deflate. The pressure *P*_*atm*_ is atmospheric pressure. Between each node in the system there is resistance to flow, *R*_*i*_, and airflow, *q*_*i*_. Blue arrows represent the flow during inspiration, and green arrows represent the flow during expiration. The grey shaded area indicates the parabronchi, where gas exchange occurs.

A schematic model of the avian respiratory system is shown in Fig 1. For simplicity, only one side of the respiratory system is shown (see Fig 12 for the full model). The caudal and cranial airsacs are considered to be flexible with lumped compliances *C*_1_ and *C*_2_ respectively and averaged pressures *P*_1_ and *P*_2_ respectively. The pressure in the coelom (thoracic-abdominal cavity) outside both sets of airsacs, *P*_*ext*_(*t*), varies periodically due to the respiratory muscles, which causes the airsacs to inflate and deflate. During inspiration (indicated by blue solid arrows in Fig 1), air flows in through the beak along the trachea (*q*_*T*_), through the primary and meso-bronchi to the caudal airsacs (*q*_1_), and from the caudal airsacs to the cranial airsacs through the parabronchi (*q*_*P*_). During expiration (indicated by green dashed arrows in Fig 1), air flows from the caudal airsacs to the cranial airsacs through the parabronchi (*q*_*P*_), from the cranial airsacs through the ventrobronchi (*q*_2_) and along the trachea to exit the beak (*q*_*T*_). The airflow pathways are considered to be rigid (no compliance) and to have resistance to airflow, *R*_*i*_, where *i* ∈ {1, 2, *T*, *P*}. Note that since our aim is to create a model that is applicable generally across all birds, and we are primarily interested in understanding the unidirectional flow through the paleopulmonic-parabronchi, we have chosen to include only the paleopulmonic-parabronchi explicitly. However, the neopulmonic-parabronchi are included indirectly in our model through the lumped resistance parameters.

From the pressures *P*_1_, *P*_2_ and *P*_*atm*_ (atmospheric pressure) and the resistances, *R*_*i*_, we can calculate the airflow, *q*_*i*_, through every pathway in the system (Eqs (17)–(20)). Using the relationship between the pressure, compliance, and volume, assuming that the compression of air is negligible, and applying some algebraic manipulations (refer to the Methods section for more details), we get the following two equations for the rate of change of the pressures *P*_1_ and *P*_2_:

$$\frac{dP_{1}}{dt}=\frac{-R_{P}R_{2}\left(P_{1}-P_{atm}\right)-\left(R_{P}R_{T}+\bar{R}\right)\left(P_{1}-P_{2}\right)}{C_{1}\bar{R}R_{P}}+\frac{dP_{ext}}{dt}$$ (1)

$$\frac{dP_{2}}{dt}=\frac{-R_{P}R_{1}\left(P_{2}-P_{atm}\right)+\left(R_{P}R_{T}+\bar{R}\right)\left(P_{1}-P_{2}\right)}{C_{2}\bar{R}R_{P}}+\frac{dP_{ext}}{dt}$$ (2)

where $\bar{R}=R_{1}R_{2}+R_{2}R_{T}+R_{T}R_{1}$.

**Fig 2:**
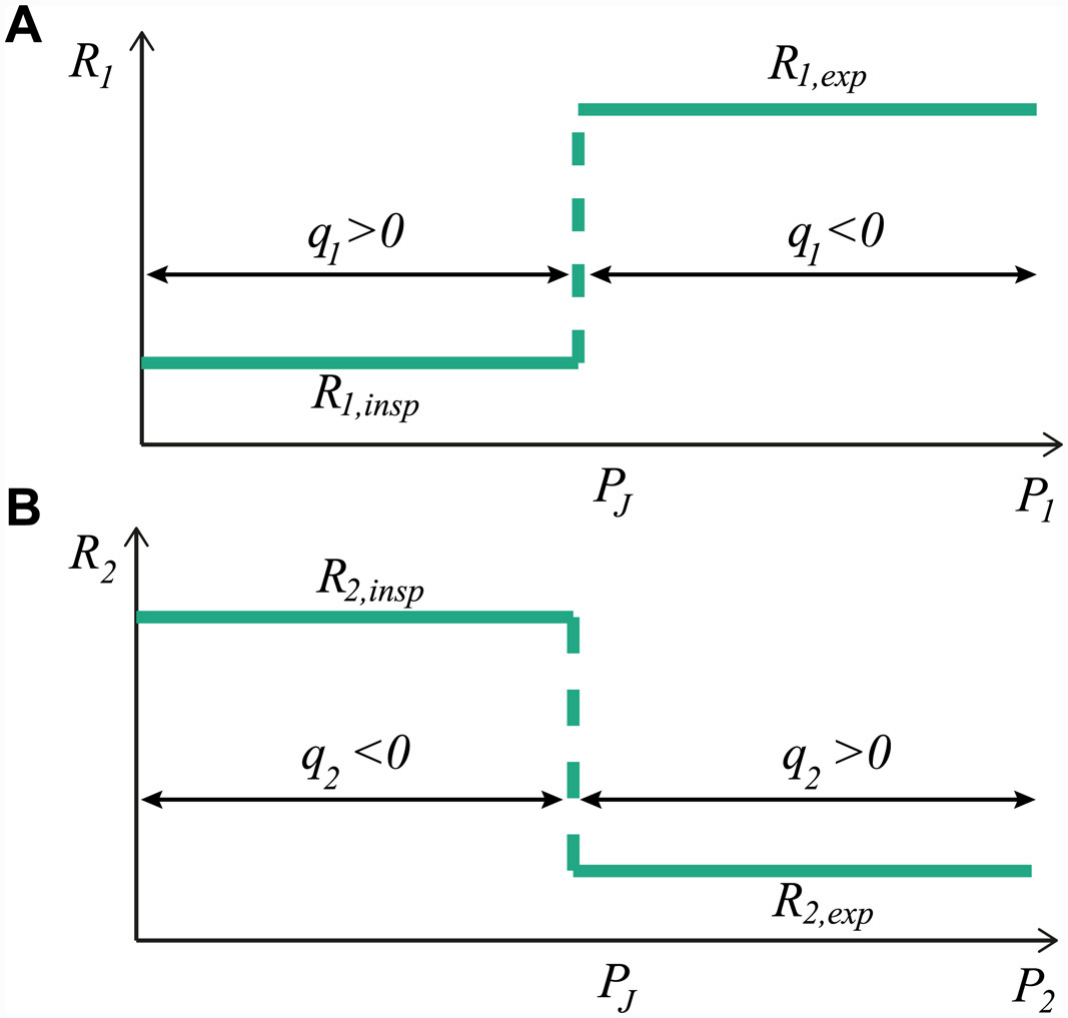
The resistances *R*_1_ and *R*_2_ are discontinuous and depend on the flow direction. **A:** The resistance *R*_1_ is discontinuous at *P*_1_ = *P*_*J*_, which is when *q*_1_ = 0. For *q*_1_ > 0, *R*_1_ = *R*_1,*insp*_, while for *q*_1_ < 0, *R*_1_ = *R*_1,*exp*_ ≥ *R*_1,*insp*_. **B:** The resistance *R*_2_ is discontinuous at *P*_2_ = *P*_*J*_, which is when *q*_2_ = 0. For *q*_2_ > 0, *R*_2_ = *R*_2,*exp*_, while for *q*_2_ < 0, *R*_2_ = *R*_2,*insp*_ ≥ *R*_2,*exp*_.

The resistances *R*_1_ and *R*_2_ are discontinuous and vary depending on the flow direction as shown in Fig 2. This allows us to produce effective inspiratory and expiratory valving:

- *Inspiratory valving*. During inspiration, it is observed that air flows through the junction *P*_*J*_ into the caudal airsacs (*q*_1_ ≈ *q*_*T*_), with very little fresh air flowing into the cranial airsacs (*q*_2_ ≪ *q*_*T*_). This is attributed to anatomical features including the T-shape of the junction, the narrowing of the airway at the segmentum accelerans, and the branching nature of the airway tree [2, 14, 29, 32–36]. We incorporate this valving effect into our model by increasing *R*_2_ during inspiration (see Fig 2B) and we measure the effectiveness of the valving by calculating how much of the flow into the animal flows through to the caudal airsacs during inspiration (labeled INSP).
  $$\text{Inspiratory}\;\;\text{valving}\;\;\text{efficacy}=\frac{\int_{\text{INSP}}{\text{q}}_{1}\;\text{dt}}{\int_{\text{INSP}}{\text{q}}_{\text{T}}\;\text{dt}}$$ (3)
- *Expiratory valving*. During expiration, it is observed that most of the air from the caudal airsacs flows through the parabronci into the cranial airsacs (*q*_*P*_), with very little fresh air flowing back without undergoing gas exchange (*q*_1_ ≪ *q*_*T*_). Expiratory valving is thought to be due to the specific anatomical structure and alignment of airsacs and dorsobronchial airways [37] but is not as well studied as the inspiratory valving. We incorporate this valving into our model by increasing *R*_1_ during expiration (see Fig 2A) and we define the effectiveness of the valving as how much of the flow out of the system comes from the cranial airsacs during expiration (labeled EXP).
  $$\text{Expiratory}\;\;\text{valving}\;\;\text{efficacy}=\frac{\int_{\text{EXP}}{\text{q}}_{2}\;\text{dt}}{\int_{\text{EXP}}\left(-{\text{q}}_{\text{T}}\right)\;\text{dt}}$$ (4)
  Note that other definitions of expiratory valving efficacy exist in the literature, see Eq (49).

### Model outputs

**Fig 3:**
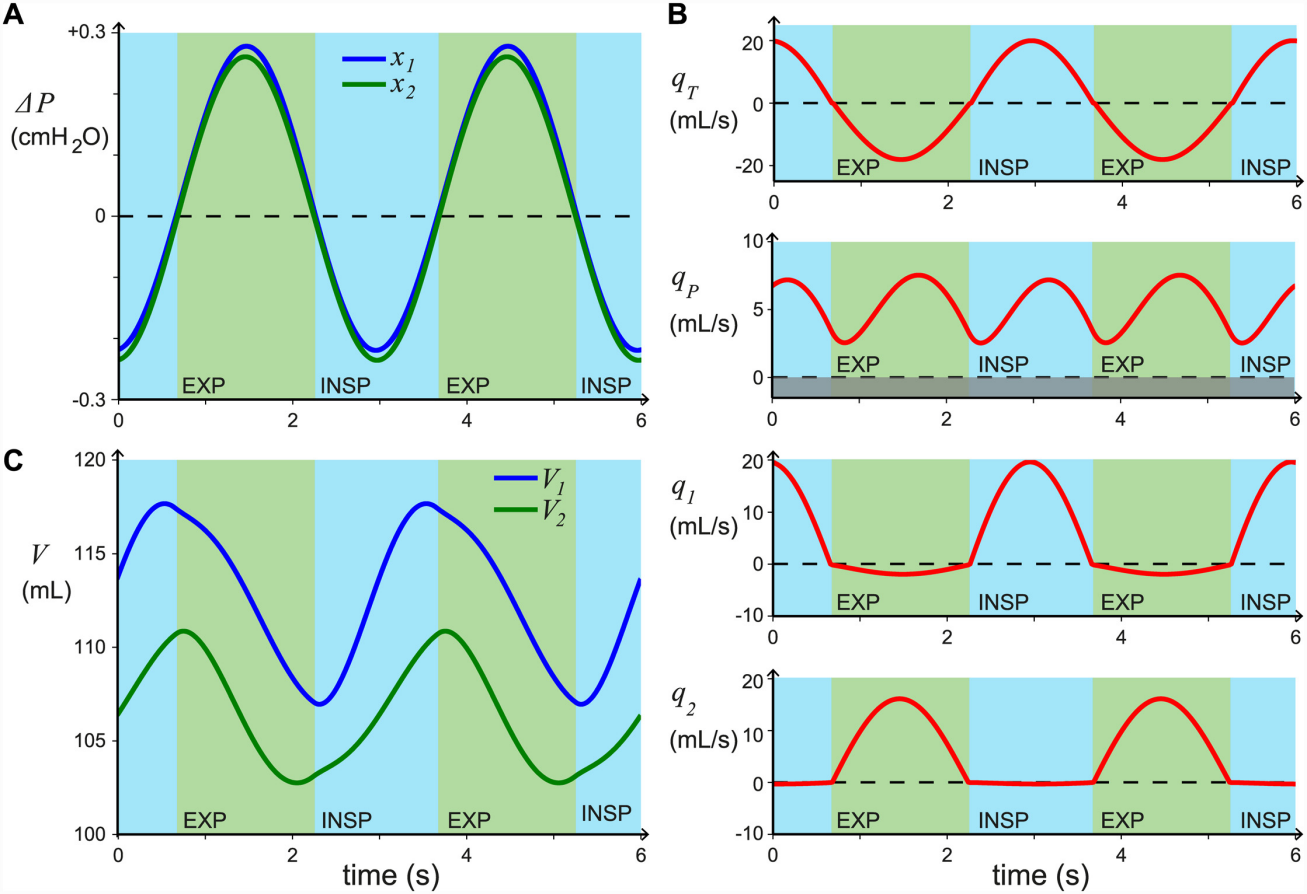
Model outputs for the chosen default parameter values (Table 1). **A:** the pressure differences from atmospheric pressure, *x*_1_ = *P*_1_ − *P*_*atm*_ and *x*_2_ = *P*_2_ − *P*_*atm*_, which are the outputs of the model. **B:** the flow rates *q*_*T*_, *q*_*P*_, *q*_1_, and *q*_2_ in one side of the respiratory system. **C:** the volumes of the caudal set of airsacs, *V*_1_, and the cranial set of airsacs, *V*_2_, on one side of the respiratory system. Inspiration and expiration phases are labelled (INSP and EXP).

A representative example of the results found in this model is shown in Fig 3, with the parameter values listed in Table 1. In Fig 3A we see that the pressure differences between the airsacs and atmospheric pressure (*x*_1_ = *P*_1_ − *P*_*atm*_ and *x*_2_ = *P*_2_ − *P*_*atm*_) are orders of magnitude greater than the difference between the two airsac pressures (*x*_1_ − *x*_2_). This matches experimental measurements, e.g. [12, 15]. Fig 3B shows that the flow through the parabronchi is unidirectional (*q*_*P*_ > 0) and that the valving is working well as *q*_2_ ≈ 0 during inspiration and *q*_1_ ≈ 0 during expiration. The tidal volume is 36.0 mL and the combined flow through the parabronchi per breath is 31.3 mL on both sides, so most of the air which is breathed in passes through the gas exchange area.

**Table 1:** Model parameters and default values used in the numerical calculations.

Parameter	Meaning	Value and Units
*P*_*atm*_	atmospheric pressure	1033.6 cmH_2_O
*R*_*trachea*_	tracheal resistance	1 cmH_2_O/L⋅s
*R*_*EPPB*_	resistance of extrapulmonary primary bronchi (EPPB) including segmentum accelerans	8 cmH_2_O/L⋅s
*R*_T_*	effective tracheal and primary bronchus resistance	10 cmH_2_O/L⋅s
*R*_*P*_	dorsobronchi and parabronchial resistance	2.5 cmH_2_O/L⋅s
*R*_1,*insp*_	mesobronchus resistance during inspiration	1 cmH_2_O/L⋅s
*R*_2,*insp*_ ^†^	effective ventrobronchi resistance during inspiration	100 cmH_2_O/L⋅s
*R*_1,*exp*_ ^†^	effective mesobronchus resistance during expiration	50 cmH_2_O/L⋅s
*R*_2,*exp*_	ventrobronchi resistance during expiration	5 cmH_2_O/L⋅s
*γ*	ratio of caudal to cranial airsac compliance	1.35
*C*_*tot*_	total parallel compliance of caudal and cranial airsacs	450 mL/cmH_2_O
*C*_1_ ^‡^	lumped compliance of caudal airsacs	258.5 mL/cmH_2_O
*C*_2_ ^‡^	lumped compliance of cranial airsacs	191.5 mL/cmH_2_O
*V*_1,*res*_	resting volume of the caudal airsacs	105.6mL
*V*_2,*res*_	resting volume of the cranial airsacs	103.6mL
*P*_*c*_	baseline pressure in the coelom (thoracic-abdominal cavity)	1033.6 cmH_2_O
*P*_*amp*_	peak-to-peak amplitude of pressure variation due to breathing	0.5 cmH_2_O
*T*	respiratory period	3 s

* *R*_*T*_ is calculated from *R*_*trachea*_ and *R*_*EPPB*_, and will change if *R*_*trachea*_ and *R*_*EPPB*_ are not the default values.

^†^ *R*_2,*insp*_ and *R*_1,*exp*_ are calculated from *R*_1,*insp*_ and *R*_2,*exp*_ respectively and depend on the strength of effective valving. If the strength of valving changes, or *R*_1,*insp*_ and *R*_2,*exp*_ are not the default values, *R*_2,*insp*_ or *R*_1,*exp*_ will change.

^‡^ *C*_1_ and *C*_2_ are calculated from *C*_*tot*_ or *γ*, and will change if *C*_*tot*_ or *γ* are not the default values.

Fig 3C shows the volumes of the caudal set of airsacs, *V*_1_, and the cranial set of airsacs, *V*_2_, on one side of the respiratory system (see also Fig 12). The volumes can be calculated directly from Eqs (9) and (10). The ventilation volume into each set of airsacs is calculated using max(*V*_*i*_)-min(*V*_*i*_) for *i* = 1, 2 and is independent of the chosen parameters *P*_*c*_, and *V*_*i*,*res*_. For the results shown in Fig 3 the ventilation volume is found to be 21.5 mL per breath in total for the caudal airsacs, on both sides, and 16.3 mL per breath in total for the cranial airsacs, on both sides. These values match experimental data [38]. Note that the sum of the ventilation of all the airsacs can be greater than or less than the tidal volume, as some air flows past the airsacs, and some air flows into both sets of airsacs.

### Conditions for unidirectional airflow

By analysing the phase plane dynamics of the system of Eq (1) (see Methods section), we can show that airflow through the parabronchi will be unidirectional (*q*_*P*_ > 0) when *γR*_1_ ≤ *R*_2_ during inspiration and *γR*_1_ ≥ *R*_2_ during expiration, where *γ* = *C*_1_/*C*_2_. In the borderline case, where *γR*_1_ = *R*_2_ during both inspiration and expiration, the flow through the parabronchi, *q*_*P*_, is zero. A combination of effective inspiratory and expiratory valving (*γR*_1_ < *R*_2_ during inspiration and *γR*_1_ > *R*_2_ during expiration) will produce unidirectional flow. However, unidirectional flow could also be achieved by inspiratory or expiratory valving alone, e.g. effective inspiratory valving, where *γR*_1_ < *R*_2_ during inspiration and *γR*_1_ = *R*_2_ during expiration, or effective expiratory valving, where *γR*_1_ = *R*_2_ during inspiration and *γR*_1_ > *R*_2_ during expiration.

**Fig 4:**
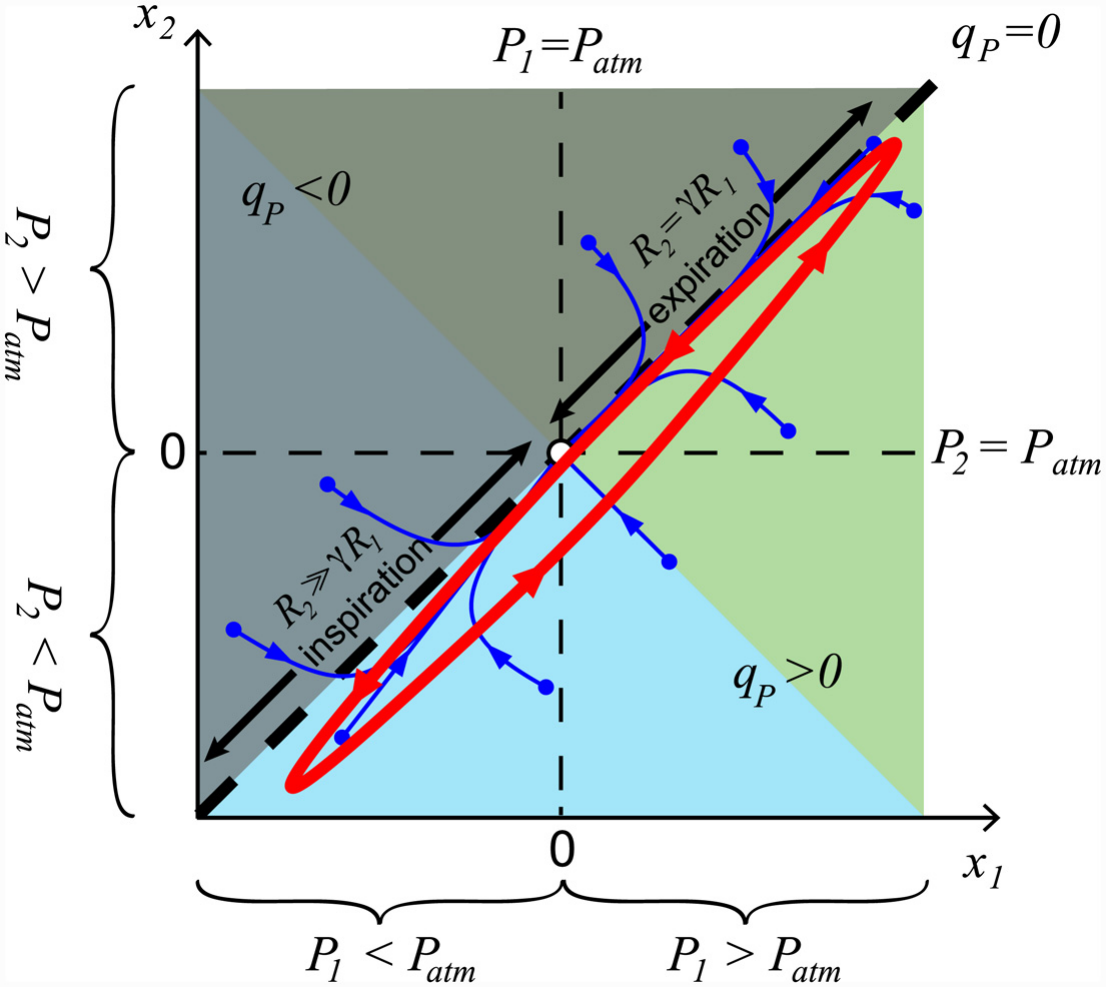
Sketch of the system dynamics (not to scale), showing conditions for unidirectional flow when there is effective inspiratory valving. The variables are *x*_1_ = *P*_1_ − *P*_*atm*_ and *x*_2_ = *P*_2_ − *P*_*atm*_. The line *x*_2_ = *x*_1_ marks all the possible pressures for which the flow *q*_*P*_ = 0. Above this line *q*_*P*_ < 0 (marked in shaded grey), and below this line *q*_*P*_ > 0. Inspiration is marked by the light blue region and expiration is marked by the green region. Dark blue curves show the solutions to the system from different initial conditions, when the external pressure is constant. Superimposed in red is an example of a solution to the system when the external pressure changes (along the vector [1, 1]^*T*^) due to the respiratory muscles during breathing. This solution is ‘trapped’ below the line *x*_1_ = *x*_2_ in the region *q*_*P*_ > 0 and hence the flow is unidirectional. See Methods and Fig 13 for a detailed analysis.

Fig 4 shows a sketch of the phase plane dynamics in the case of effective inspiratory valving, where the variables are transformed such that *x*_1_ = *P*_1_ − *P*_*atm*_ and *x*_2_ = *P*_2_ − *P*_*atm*_. The stable equilibrium *P*_1_ = *P*_2_ = *P*_*atm*_ in the absence of pressure variations (no breathing) is then at the origin (0, 0). The line *x*_2_ = *x*_1_ marks all the possible pressures for which the flow *q*_*P*_ is zero. Above this line (*x*_2_ > *x*_1_) *q*_*P*_ is negative (marked in shaded grey), and below it (*x*_2_ < *x*_1_) *q*_*P*_ is positive. The dark blue curves show the solutions to the system from different initial conditions when there is no breathing, and thus no change in the external pressure. All these solutions approach the origin (0, 0). The red curve shows the solution (not to scale) of the system when the external pressure *P*_*ext*_ is changing due to breathing. This change in pressure is the same outside both sets of airsacs and thus acts along the vector [1, 1]^*T*^. It can be seen that the system is ‘trapped’ below the line *x*_1_ = *x*_2_ and therefore the airflow is unidirectional (see Methods section for more details).

In Fig 4 inspiration is marked by the light blue region and expiration is marked by the green region. When both *P*_1_ and *P*_2_ are greater than *P*_*atm*_ (upper right quadrant) it is obvious that expiration will occur (*q*_*T*_ < 0). When both *P*_1_ and *P*_2_ are less than *P*_*atm*_ (lower left quadrant), inspiration will occur (*q*_*T*_ > 0). The exact place (in the lower right quadrant) where the transition from inspiration to expiration occurs in the phase plane will depend on the chosen parameter values. Specifically, the flow *q*_*T*_ will change direction on the line $x_{2}=-\frac{R_{2,insp}}{R_{1,exp}}x_{1}$ with inspiration occurring if $x_{2}<-\frac{R_{2,insp}}{R_{1,exp}}x_{1}$ and expiration occurring if $x_{2}>-\frac{R_{2,insp}}{R_{1,exp}}x_{1}$ (see Methods section and Fig 13). For simplicity, in Fig 4 we consider the case where *R*_2,*insp*_ = *R*_1,*exp*_ and the transition between inspiration and expiration occurs on the line *x*_2_ = −*x*_1_.

### Unidirectional flow is robust to changes in the breathing amplitude and frequency

**Fig 5:**
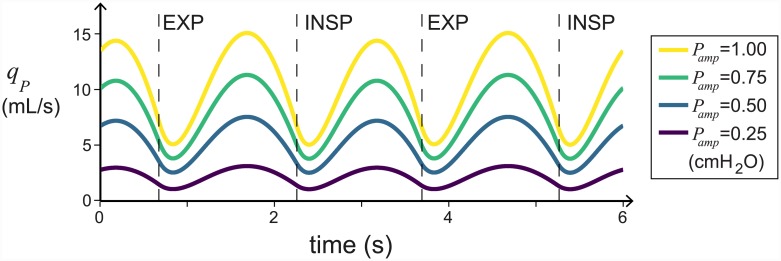
Unidirectional flow is robust to changes in amplitude. In this figure we plot the flow through the parabronchi, *q*_*P*_, against time for a range of *P*_*amp*_ values. The flow rates increase linearly as the amplitude of breathing, *P*_*amp*_ increases. Inspiration and expiration are labelled (INSP and EXP). All parameters, except *P*_*amp*_, are as in Table 1.

**Fig 6:**
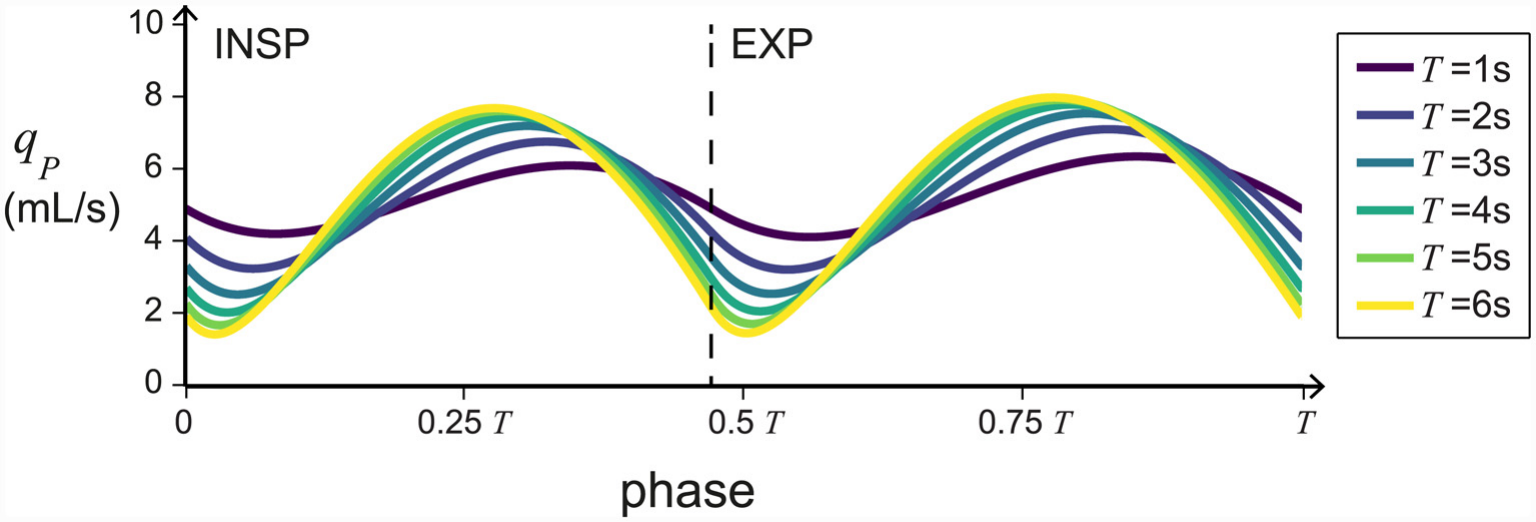
Unidirectional flow is robust to changes in frequency. The flow through the parabronchi, *q*_*P*_, during a single breath, is plotted for breathing period, *T*, ranging from 1–6 seconds (frequency ranging from 1–1/6 Hz respectively). The traces are aligned such that phase = 0 is at the beginning of inspiration. Inspiration and expiration are labelled (INSP and EXP). All the parameters, except the breathing period, are as in Table 1.

The conditions for unidirectional flow do not depend on the frequency or amplitude of breathing. Consequently, unidirectional flow will persist as long as the conditions on the resistances and compliances (stated in the previous section) are satisfied. Increasing the amplitude, *P*_*amp*_, increases the flow rates proportionally (see Fig 5). As the period, *T*, decreases (the frequency increases) the mean flow through the parabronchi remains relatively constant, but the variation in the flow rate during the breathing cycle decreases and the flow becomes more constant (see Fig 6). This matches what is seen experimentally [11], and may have an impact on the efficacy of gas exchange.

### Efficient airflow requires two effective valves

In the avian system, the aerodynamic valving is not 100% effective. Inspiratory valving is found experimentally to be 95–100% effective [14, 32–34, 39]. However, expiratory valving efficacy varies greatly between species and experimental conditions: 76–90% in chickens [12, 40], 88% in ducks [14], and 95% in geese [37], with a strong dependence on gas velocity; at higher flow rates (exercise conditions) the valve is more effective than at rest.

When fresh air flows into the cranial or caudal airsacs and is then breathed back out without passing through the parabronchi, this air does not undergo gas exchange, and is thus wasted. We define the *efficiency* of the whole system as the fraction of the tidal volume that passes through the parabronchi. For example, when efficiency = 1 all the air that is inhaled will pass through the parabronchi and undergo gas exchange. We calculate the efficiency in our model by numerically integrating the airflow *q*_*P*_ during one cycle of breathing to find the total volume of air that flows through the parabronchi per breath, and numerically integrating the flow *q*_*T*_ during inspiration to calculate the tidal volume (the total air inhaled per breath). The ratio of volume through parabronchi per breath to tidal volume gives us a measure of how efficient the lung system is.

$$\text{Efficiency}=\frac{\int_{\text{INSP}+\text{EXP}}{\text{q}}_{\text{P}}\;\text{dt}}{\int_{\text{INSP}}{\text{q}}_{\text{T}}\;\text{dt}}$$ (5)

where ∫_*INSP*_ indicates the definite integral during inspiration and ∫_*INSP*+*EXP*_ indicates the definite integral during one breath.

**Fig 7:**
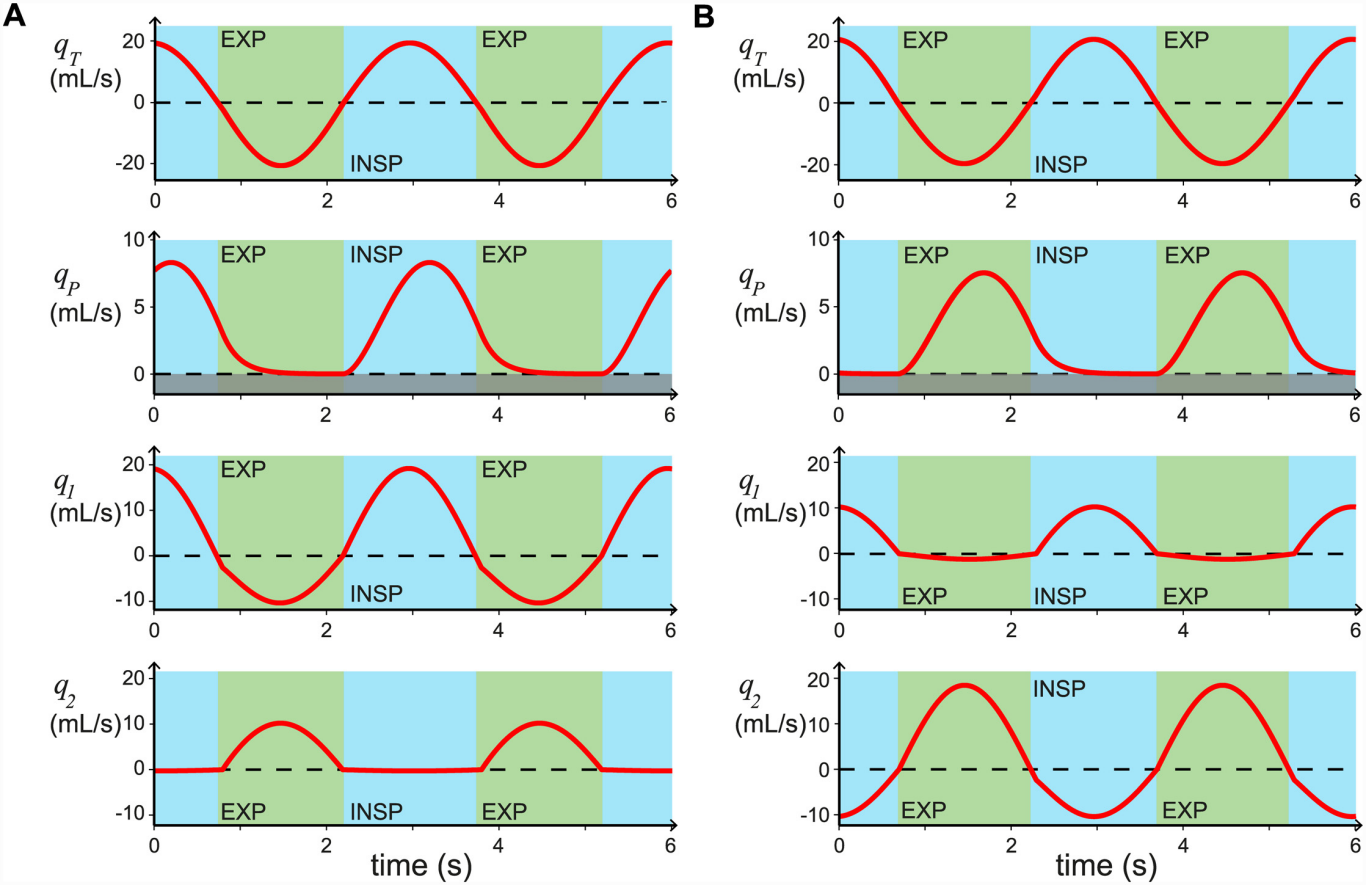
Inspiratory and expiratory valving both produce unidirectional flow, *q*_*P*_ > 0. Flow rates *q*_*T*_, *q*_*P*_, *q*_1_, and *q*_2_ against time for the parameters *R*_1,*insp*_ = *R*_2,*exp*_ = 3 cmH_2_O/L⋅s, *C*_1_ = *C*_2_ (*γ* = 1), and *C*_*tot*_ = 450 mL/cmH_2_O. Panel **A** shows the case where there is effective inspiratory valving: *R*_2,*exp*_ = *γR*_1,*exp*_ and *R*_2,*insp*_ = 100 × *R*_1,*insp*_, with *R*_1,*exp*_ = *R*_1,*insp*_. Panel **B** shows the case with effective expiratory valving: *R*_1,*insp*_ = *γR*_2,*insp*_ and *R*_1,*exp*_ = 20 × *R*_2,*exp*_, with *R*_2,*insp*_ = *R*_2,*exp*_. All other parameters are given in Table 1.

If the model includes effective inspiratory valving only (*R*_2,*insp*_ ≫ *γR*_1,*insp*_ and *R*_2,*exp*_ = *γR*_1,*exp*_) with *R*_1,*insp*_ = *R*_1,*exp*_, the flow *q*_*P*_ is unidirectional (*q*_*P*_ > 0), but the maximum efficiency we can find numerically is around 50%. The cause of this low efficiency is that a large proportion of the fresh air that flows into the caudal airsacs, then flows back out (*q*_1_ < 0) without participating in gas exchange, as shown in Fig 7A. In Fig 7A, the inspiratory valving efficacy = 98.7%, the expiratory valving efficacy = 47.7%, the overall efficiency is 47.1%, the tidal volume is 38.1 mL whilst the flow through both parabronchi per breath is only 17.9 mL.

Similarly, if the model includes only effective expiratory valving (*γR*_1,*insp*_ = *R*_2,*insp*_ and *γR*_1,*exp*_ ≫ *R*_2,*exp*_) with *R*_2,*insp*_ = *R*_2,*exp*_, we find numerically that the maximum efficiency we can reach is around 50%, due to flow into the cranial airsacs during inspiration (*q*_2_ < 0). An example of effective expiratory valving is shown Fig 7B, where the inspiratory valving efficacy is 48.6% and the expiratory valving efficacy is 87.0%. This gives an overall efficiency of 42.3%; from a tidal volume of 38.4 mL and only 16.2 mL flow through both parabronchi per breath.

By including both inspiratory and expiratory valving we find that we can reduce this back flow, and when we match the experimental valving efficiencies of 98–100% for inspiratory valving and ≈88% for expiratory valving, we find that the overall efficiency of the system matches those found experimentally, whilst maintaining realistic resistance and compliance values. The flow rates for our chosen parameter values are shown in Fig 3B, where the inspiratory valving efficacy is 98.0%, the expiratory valving efficacy is 88.6%, and the overall efficiency is 86.8%.

### Efficiency is affected more by asymmetries in the resistance to flow than by the compliances

**Fig 8:**
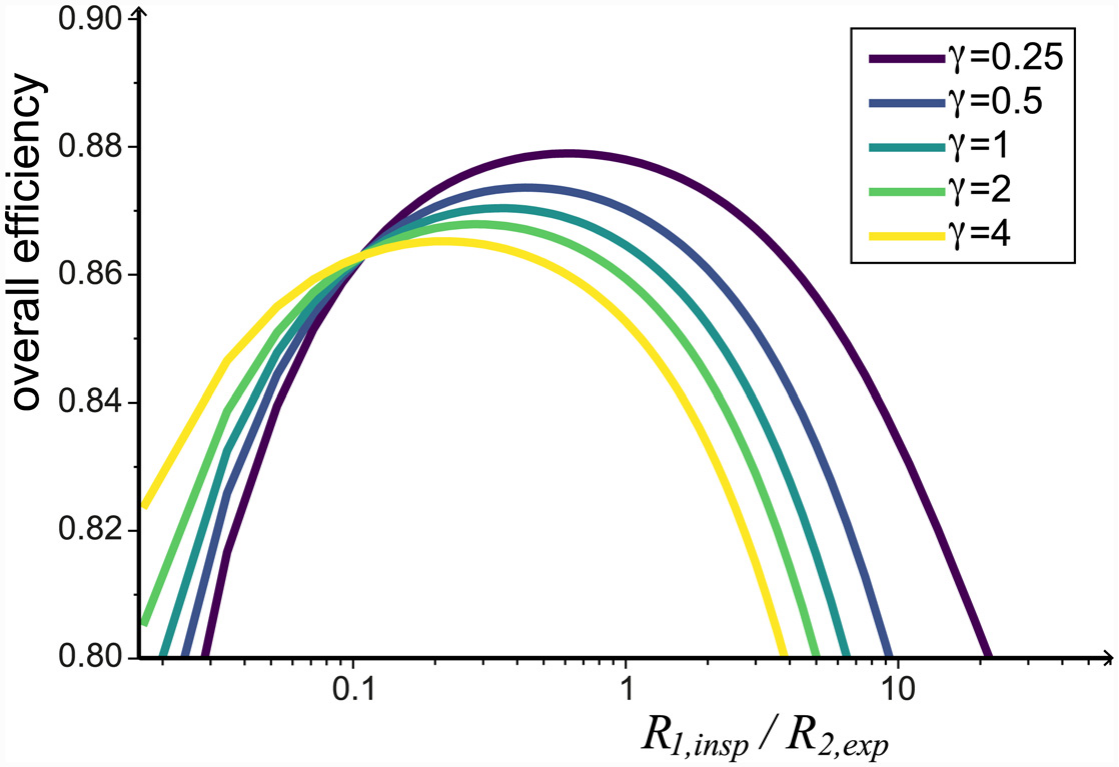
Changing the relative resistance of *R*_1,*insp*_ / *R*_2,*exp*_ affects the efficiency of the system. Plot of the overall efficiency when *R*_1,*insp*_ / *R*_2,*exp*_ is varied whilst keeping the total resistance of the system constant (*R*_1,*insp*_ + *R*_2,*exp*_ = 6 cmH_2_O/L⋅s). The effect is similar for a range of *γ* values. All other parameters are given in Table 1.

In our model, we can investigate the impact of varying the resistances *R*_1,*insp*_ and *R*_2,*exp*_. We need to keep *R*_1,*insp*_ + *R*_2,*exp*_ constant so that there is no change in the total resistance of the system, here we choose *R*_1,*insp*_ + *R*_2,*exp*_ = 6 cmH_2_O/L⋅s. Additionally, it is important to keep *R*_2,*insp*_ = 100 × *R*_1,*insp*_ and *R*_1,*exp*_ = 10 × *R*_2,*exp*_, so that the strength of the valving isn’t changing. Fig 8 shows that depending on the ratio of compliances, *γ*, the maximum efficiency will be for 0.2 < *R*_1,*insp*_/*R*_2,*exp*_ < 1. This is consistent with experimental observations that found *R*_1,*insp*_ to be lower than *R*_2,*exp*_ (see Methods section). Comparatively, we find that varying *γ* and *C*_*tot*_ affect the efficiency by less than 1% (see Selecting model parameters).

### The timing of the airflow through the parabronchi depends on the relative airsac compliance, *γ*

The airflow through the parabronchi is not constant during the breathing cycle. An important feature of the flow through the parabronchi is that it can be observed to occur mostly during inspiration, or expiration, or both, depending on parameter values and experimental conditions [10, 11].

From Fig 7 we can see that the aerodynamic valving affects the timing of the airflow through the parabronchi; effective inspiratory valving increases parabronchial flow during inspiration (Fig 7A), whereas effective expiratory valving increases parabronchial flow during expiration (Fig 7B). However, once we fix the valving efficacy to physiologically realistic levels (e.g. inspiratory valving 98%, expiratory valving 88%), the ratio of volume flowing through the parabronchi during inspiration and expiration (I:E parabronchial volume flow ratio) due to the valving is fixed. We calculate the ratio of volume flowing through the parabronchi during inspiration and expiration from the output of our model as follows:

$$I:E\;\;\text{parabronchial}\;\;\text{volume}\;\;\text{flow}\;\;\text{ratio}=\frac{\int_{\text{INSP}}{\text{q}}_{\text{P}}\;\text{dt}}{\int_{\text{EXP}}{\text{q}}_{\text{P}}\;\text{dt}}$$ (6)

For the default parameter values (Table 1) the I:E parabronchial volume flow ratio is 0.862, i.e. there is slightly less flow during inspiration than during expiration, as shown in Fig 3.

**Fig 9:**
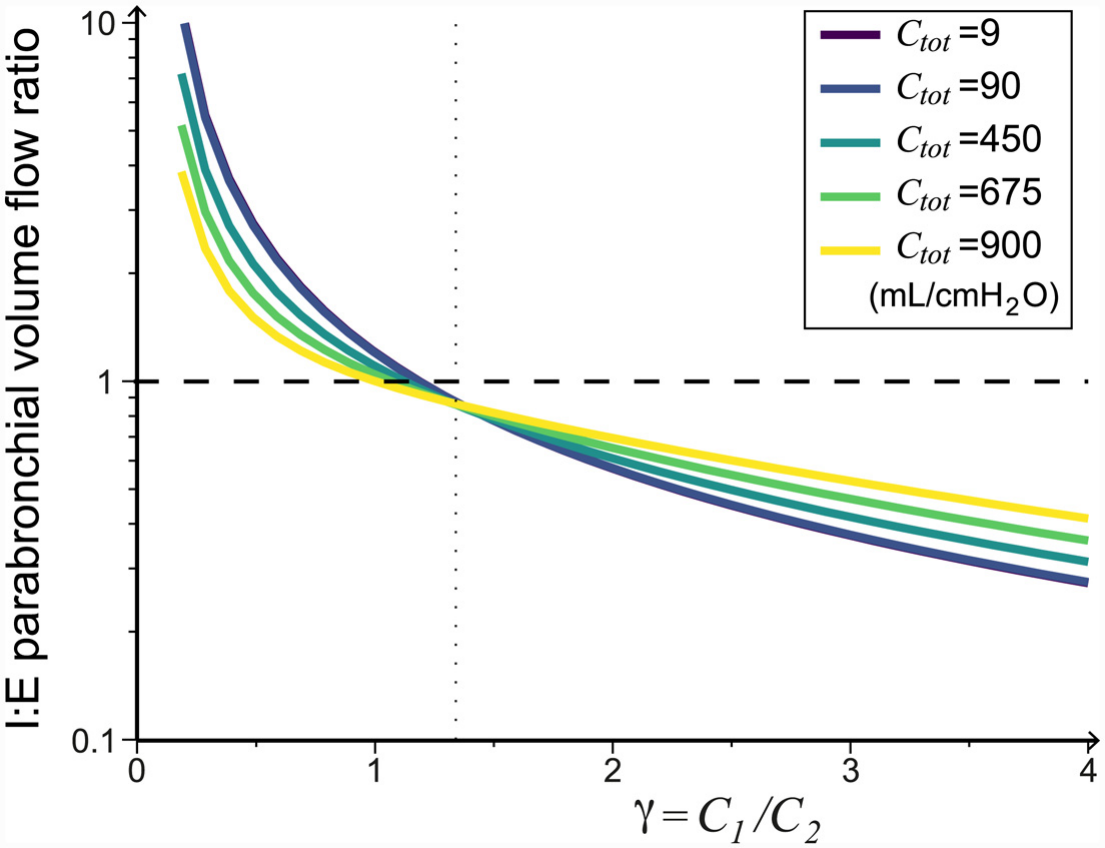
Varying the ratio of compliances *γ* = *C*_1_/*C*_2_ changes the timing of the flow through the parabronchi. As *γ* increases (*C*_1_ increases relative to *C*_2_) more air flows through the parabronchi during expiration. The dashed line at 1 indicates when the total flow during expiration and inspiration are equal. All the parameters are as given in Table 1. The vertical dotted line shows the selected default *γ* value.

Varying *γ* has a major impact on the timing of the flow through the parabronchi (recall that *γ* = *C*_1_/*C*_2_). When *γ* is low the majority of the flow through the parabronchi occurs during inspiration, while when *γ* is high the flow through the parabronchi occurs mostly during expiration. In Fig 9 we plot the I:E parabronchial volume flow ratio as a function of *γ*. As *γ* increases we find that the majority of the flow *q*_*P*_ moves from being during the inspiratory phase to being during the expiratory phase. This result is conserved for a range of total compliance (*C*_1_ + *C*_2_ = *C*_*tot*_) values. Despite this change in the timing of the flow, the system’s overall efficiency only decreases slightly (from 87.7% to 86.4%) when *γ* increases (see S1A Fig), and the airflow through the parabronchi remains unidirectional.

**Fig 10:**
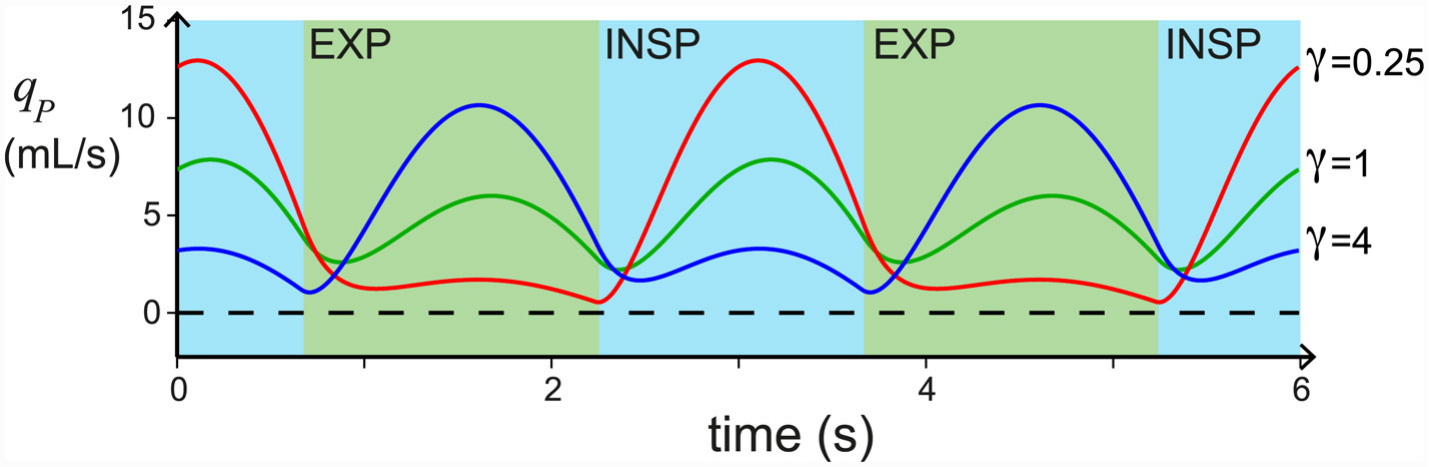
The ratio of compliances *γ* = *C*_1_/*C*_2_ changes the shape of the oscillatory flow *q*_*P*_. This figure plots the flow rate *q*_*P*_ versus time, for *γ* = 1/4 (red), *γ* = 1 (green), and *γ* = 4 (blue). The inspiratory period (INSP) is shaded blue, and the expiratory period (EXP) is shaded green. All other parameters are as given in Table 1.

These results are consistent with experimental observations. Anatomically, the caudal and cranial airsacs are found to have different properties [2]. In ducks, Scheid et al. [38] found that the caudal airsacs are more compliant and have larger ventilation volume changes than the cranial airsacs, especially during relaxed (anaesthetized) breathing. Furthermore, the ratio of compliances varies between individuals and species [1] and many variations in the flow pattern are also observed [10, 11]. For example, in spontaneously breathing geese the flow through the parabronchi increases at the end of inspiration and peaks during expiration [10]. Looking at ducks it is found that the flow during inspiration is higher than the flow during expiration when panting, the flow during inspiration and expiration are similar in spontaneous breathing, and when relaxed (anaesthetized) the flow rate is much stronger during expiration [11]. Our results as we vary *γ* provides similar changes in flow patterns (Fig 10), which agree with experimental findings; *γ* should decrease during exercise when the abdominal and chest muscles are stiff, and increase under relaxation conditions when the muscles relax.

We find that changing the relative resistances *R*_1,*insp*_/*R*_2,*exp*_ does affect the timing of the flow through the parabronchi slightly, with more flow during expiration as *R*_1,*insp*_/*R*_2,*exp*_ increases (see S2A Fig). We also find that varying the total compliance *C*_*tot*_ does not change the timing of the flow through the parabronchi substantially, but the strength of the effect decreases at high *C*_*tot*_ (see S2B Fig). Overall, the ratio of compliances, *γ*, is the dominant effect.

### The durations of inspiration and expiration depend primarily on the relative resistances *R*_1,*insp*_ and *R*_2,*exp*_

We observe that although the forcing of the system is symmetric (sinusoidal function), the duration of inspiration, *T*_*i*_ (measured as the time during which *q*_*T*_ > 0), is not always equal to the duration of expiration, *T*_*e*_ (measured as the time during which *q*_*T*_ < 0). For our chosen default parameter values (Table 1), with period *T* = 3s, *T*_*i*_ = 1.4s and *T*_*e*_ = 1.6s, and the ratio of inspiration duration to expiration duration (I:E time ratio = *T*_*i*_/*T*_*e*_) is 0.89. This asymmetry varies depending on parameter values.

**Fig 11:**
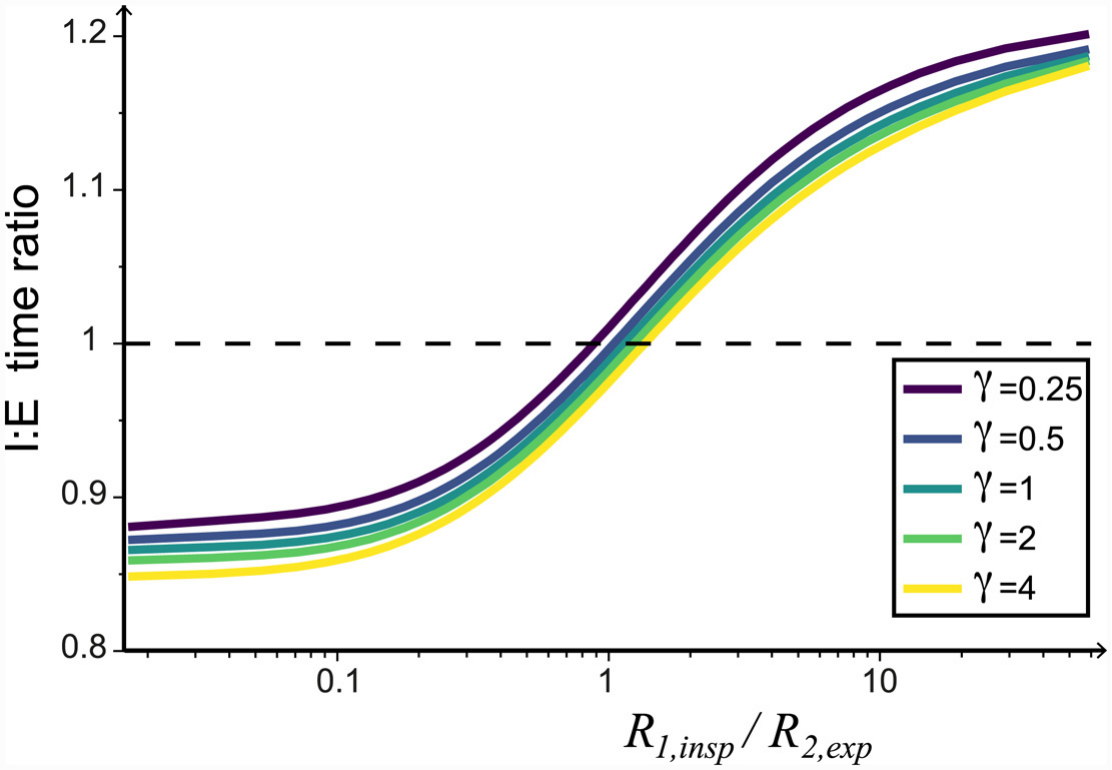
The relative resistance of *R*_1,*insp*_/*R*_2,*exp*_ affects the duration of the expiration and inspiration phases. Here we plot the ratio of the inspiration and expiration phase durations (I:E time ratio) against the relative resistance of *R*_1,*insp*_/*R*_2,*exp*_ whilst keeping the total resistance constant (*R*_1,*insp*_ + *R*_2,*exp*_ = 6 cmH_2_O/L⋅s). The same effect is seen for a range of *γ* values. The dashed line indicates where the period of expiration and inspiration are equal, *T*_*e*_ = *T*_*i*_.

We investigate the impact of varying the resistances *R*_1,*insp*_ and *R*_2,*exp*_, while the overall resistance of the system and the strength of the valving constant, as before. We find that when we decrease *R*_1,*insp*_ relative to *R*_2,*exp*_ the duration of expiration increases, with a concordant decrease in the duration of inspiration (Fig 11).

The ratio of compliances, *γ*, and the total compliance, *C*_*tot*_, do affect the I:E time ratio slightly, but the impact of varying the relative resistances is much stronger (see Selecting model parameters).

## Discussion

We have constructed a relatively simple mathematical model of the avian respiratory system which, for the first time, produces unidirectional airflow through the parabronchi that is robust to changes in breathing frequency, breathing amplitude, and model parameters. Using physiologically reasonable parameters (in this case we use those found for ducks) the model produces efficiencies, flow rates, and pressures that match experimental findings.

It has been hypothesized that the unidirectional flow is due to inspiratory and expiratory aerodynamic valves, resulting from the anatomical structure and the fluid dynamics involved. We incorporated these aerodynamic valves into our model by increasing the resistance to flow from the primary bronchus to the cranial airsacs (*R*_2_ in our model) during inspiration, and increasing the resistance to flow from the caudal airsacs to the primary bronchus (*R*_1_ in our model) during expiration. We showed that both of these resistances as well as the airsac compliances (*C*_1_ and *C*_2_ in our model) affect the efficacy of the inspiratory and expiratory valving. This result could explain why models that focused on limited areas of the respiratory system or that oversimplified the pathways’ geometry were unable to produce the valving [29, 30]. We further showed that unidirectional flow could be produced by either an effective inspiratory or an effective expiratory valve (Fig 7), but that both inspiratory and expiratory valves are required to produce the high efficiencies observed in avian lungs.

In existing models, the compliances of the caudal and cranial airsacs has been assumed to be the same [29, 30]. However, there is no anatomical reason why this should be the case, and indeed this is not what has been found experimentally [38]. Using our model, we varied the single parameter *γ* = *C*_1_/*C*_2_, whilst keeping the total compliance constant (*C*_*tot*_ = *C*_1_ + *C*_2_). We found that the ratio of compliances does not affect the total flow through the parabronchi significantly, but that it has a strong impact on the timing of the flow; for physiological parameter values when *C*_1_ < *C*_2_, the majority of the flow through the parabronchi occurs during inspiration, and when *C*_1_ > *C*_2_, the majority of the flow through the parabronchi occurs during expiration (Fig 9). The overall compliance of the airsacs is affected by the chest wall and muscles surrounding them. This means that the effective compliance is a parameter that could vary in different conditions, and would strongly influence the dynamics of the flow through the parabronchi and thus the gas exchange.

Our model provides additional novel insights into the operation of the avian respiratory system. We showed that changing the relative resistance of *R*_1,*insp*_ / *R*_2,*exp*_ whilst keeping the total resistance (*R*_1,*insp*_ + *R*_2,*exp*_) constant, affects the efficiency of the system and that maximum efficiencies appear to exist near physiologically realistic parameter values (Fig 8). Another interesting observation is that the ratio of *R*_1,*insp*_/*R*_2,*exp*_ affects the period of expiration and inspiration. Specifically, we found that *T*_*e*_ > *T*_*i*_ when *R*_1,*insp*_ < *R*_2,*exp*_ (Fig 11).

### Limitations

The complex anatomical structure of the avian respiratory system has been represented in our model by discontinuous resistances (*R*_1_ and *R*_2_) that depend on the direction of airflow through them. These resistance values could depend on the properties of the flow and would then vary with frequency and amplitude of breathing, as well as with other parameters such as muscle tone that we did not take into account in our model. Nevertheless, we have shown that as long as *γR*_1_ ≤ *R*_2_ during inspiration and *γR*_1_ ≥ *R*_2_ during expiration, where *γ* = *C*_1_/*C*_2_ is the ratio of the airsac compliances, unidirectional flow will persist.

We also assumed that both the inspiratory and expiratory valving are highly effective which is true during regular breathing but may not be the case if breathing consists of very high or very low frequencies or amplitudes. In particular, we note that experimentally it is found that panting and other breathing patterns (bird song/calls) do not have the same pattern. For example, during panting the expiratory valving is not strong and there is a large amount of air shunted into the primary bronchus (*q*_1_ < 0) which bypasses the parabronchi [9].

The two discontinuous resistances in our model make the system nonlinear, despite the assumptions of constant resistance and compliance elements. The presence of discontinuities in models is known to produce complicated phenomena, especially in non-autonomous systems with external forcing [41]. In this model, we have found the intriguing result that the inspiratory and expiratory periods are uneven in response to regular (sinusiodal) forcing. The phenomenon underlying this disparity is not clear yet. Further theoretical analysis is left for future investigations.

### Conclusions

In summary, the new mathematical model we have developed, and the analytical and computational study we have conducted, significantly increase our understanding of unidirectional airflow in avian lungs. Our new model is broadly applicable across all birds and can be extended or integrated into larger systems-level studies of the avian respiratory system. Our model also provides a new example of a non-smooth dynamical system and will be used in future investigations of the human respiratory system through comparative physiology.

## Methods

### Model formulation

**Fig 12:**
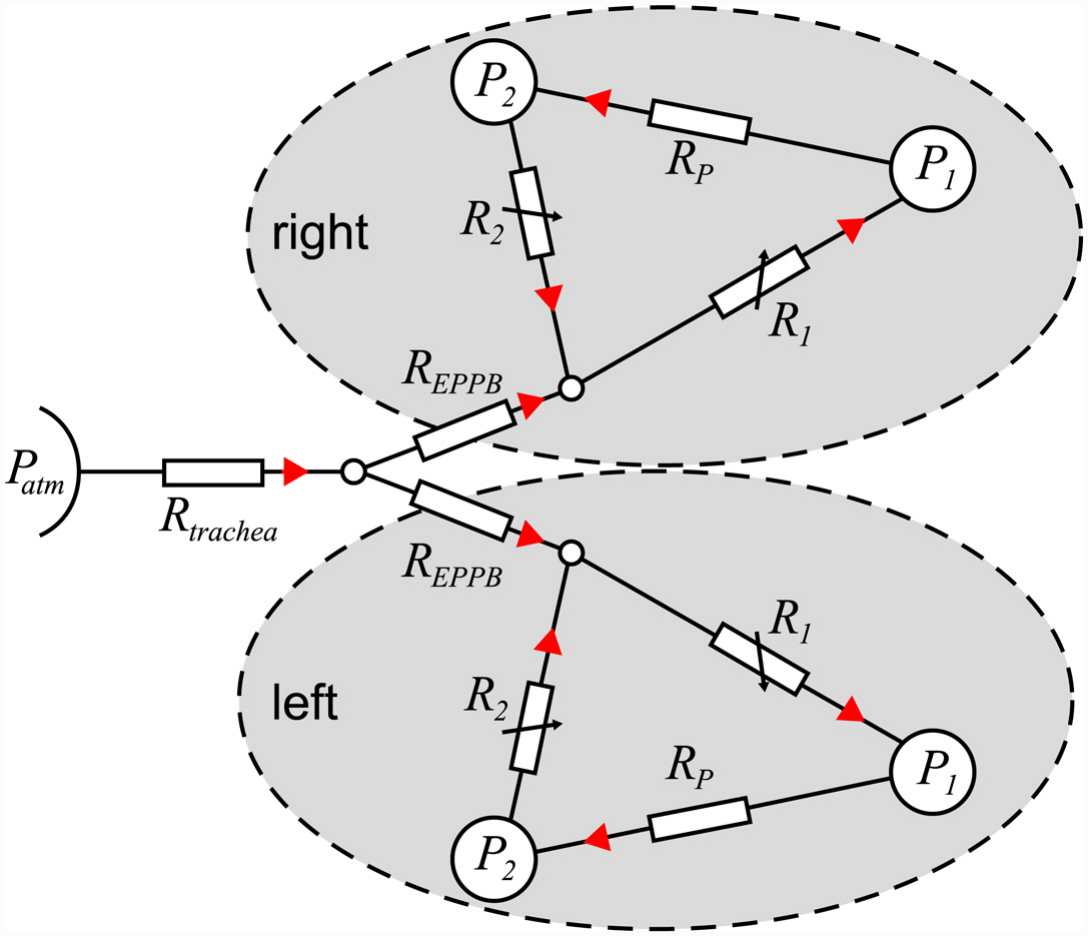
Schematic of the full avian model including left and right sides of the respiratory system. The caudal and cranial airsacs have pressures *P*_1_ and *P*_2_, respectively. The direction of positive flow rate is indicated by the red arrows. If we assume symmetry, we can reduce the model to consider only one side, which gives the model in Fig 1, with *R*_*T*_ = 2*R*_*trachea*_ + *R*_*EPPB*_. The flows found in the reduced model will be for a single side of the animal, and will need to be doubled to find the total flow in the whole animal.

Fig 12 shows the full model including left and right sides of the respiratory system. The caudal and cranial airsacs have pressures *P*_1_ and *P*_2_ respectively, and compliances *C*_1_ and *C*_2_ respectively. All other pathways and junctions are assumed to be rigid. To simplify our analysis, we assume that the left and right sides of the bird are symmetrical; this enables us to reduce the model to the system shown in Fig 1, where *R*_*T*_ = 2*R*_*trachea*_ + *R*_*EPPB*_. In the remainder of this work we will use the model shown in Fig 1, which considers only one side. To find the overall flow in the whole animal, we simply double the flow rates found from this single side.

To construct the mathematical model we begin by calculating the rate of change of volume in the caudal and cranial airsacs (*dV*_1_/*dt* and *dV*_2_/*dt*, respectively), assuming that the compression of air is negligible:

$$\frac{dV_{1}}{dt}=q_{1}-q_{P}$$ (7)

$$\frac{dV_{2}}{dt}=q_{P}-q_{2}$$ (8)

where *q*_*i*_ with *i* ∈ {1, 2, *P*} is the airflow through the corresponding section. Next we assume that the airsacs are elastic, with compliance *C*_1_ and *C*_2_. Additionally, surrounding both sets of airsacs there is an external pressure *P*_*ext*_(*t*), which has a time-varying component that represents the change in pressure generated by the muscles of the chest and abdomen during breathing. This gives the equations:

$$V_{1}=C_{1}\left(P_{1}-P_{ext}\right)+V_{1,res}$$ (9)

$$V_{2}=C_{2}\left(P_{2}-P_{ext}\right)+V_{2,res}$$ (10)

where *V*_1,*res*_ and *V*_2,*res*_ are the resting volumes of the caudal and cranial airsacs when the pressure difference between the airsacs and the surrounding thoracic-abdominal cavity (coelom) is zero. In all our simulations we use the sinusoidal function:

$$P_{ext}\left(t\right)=P_{c}-\frac{P_{amp}}{2}\cos\left(\frac{2\pi t}{T}\right)$$ (11)

to model the time-varying pressure outside the airsacs. This function oscillates with a peak-to-peak amplitude of *P*_*amp*_, which is the amplitude of the forcing from breathing, around a pressure *P*_*c*_, which is the baseline pressure in the coelom. Differentiating Eqs (9) and (10) with respect to time gives

$$\frac{dV_{1}}{dt}=C_{1}\left(\frac{dP_{1}}{dt}-\frac{dP_{ext}}{dt}\right)$$ (12)

$$\frac{dV_{2}}{dt}=C_{2}\left(\frac{dP_{2}}{dt}-\frac{dP_{ext}}{dt}\right)$$ (13)

with

$$\frac{dP_{ext}}{dt}=\frac{P_{amp}\pi }{T}\sin\left(\frac{2\pi t}{T}\right)$$ (14)

Equating Eqs (12) and (7), and Eqs (13) and (8) gives:

$$\frac{dP_{1}}{dt}=\frac{q_{1}-q_{P}}{C_{1}}+\frac{dP_{ext}}{dt}$$ (15)

$$\frac{dP_{2}}{dt}=\frac{q_{P}-q_{2}}{C_{2}}+\frac{dP_{ext}}{dt}$$ (16)

Assuming laminar flow, we obtain expressions for the flow rates *q*_*T*_, *q*_1_, *q*_2_, and *q*_*P*_ in terms of our variables *P*_1_ and *P*_2_, as well as the pressures *P*_*atm*_ and *P*_*J*_:

$$q_{T}=\frac{P_{atm}-P_{J}}{R_{T}}$$ (17)

$$q_{1}=\frac{P_{J}-P_{1}}{R_{1}}$$ (18)

$$q_{2}=\frac{P_{2}-P_{J}}{R_{2}}$$ (19)

$$q_{P}=\frac{P_{1}-P_{2}}{R_{P}}$$ (20)

From the geometry of the system, the flow at junction *J* is conserved (inflexible junction), so *q*_*T*_ + *q*_2_ = *q*_1_. From this, and flow Eqs (17)–(19) we find:

$$R_{T}\left(q_{1}-q_{2}\right)+P_{J}=P_{atm}$$ (21)

$$-R_{1}q_{1}+P_{J}=P_{1}$$ (22)

$$R_{2}q_{2}+P_{J}=P_{2}$$ (23)

Solving for *q*_1_, *q*_2_, and *P*_*J*_ in terms of *P*_1_, *P*_2_, and *P*_*atm*_ we get:

$$q_{1}=\frac{-R_{2}\left(P_{1}-P_{atm}\right)-R_{T}\left(P_{1}-P_{2}\right)}{\bar{R}}$$ (24)

$$q_{2}=\frac{R_{1}\left(P_{2}-P_{atm}\right)-R_{T}\left(P_{1}-P_{2}\right)}{\bar{R}}$$ (25)

$$P_{J}=\frac{R_{2}R_{T}P_{1}+R_{T}R_{1}P_{2}+R_{1}R_{2}P_{atm}}{\bar{R}}$$ (26)

where we introduce the combined resistance $\bar{R}=R_{1}R_{2}+R_{2}R_{T}+R_{T}R_{1}$ to simplify the equations.

Substituting the expressions for the flow rates Eqs (20), (24) and (25) into Eqs (15) and (16), we get the rate Eqs (1) and (2) for *P*_1_ and *P*_2_ respectively.

### Analysis of the model

The unique steady-state for the system of ordinary differential Eqs (1) and (2) in the absence of changing pressure due to breathing $\left(\frac{dP_{ext}}{dt}=0\right)$, is *P*_1_ = *P*_2_ = *P*_*atm*_. If we move the equilibrium point to the origin using the transformation *x*_1_ = *P*_1_ − *P*_*atm*_, *x*_2_ = *P*_2_ − *P*_*atm*_, the system of equations becomes:

$$\frac{dx_{1}}{dt}=\frac{-(R_{P}R_{T}+R_{P}R_{2}+\bar{R})}{C_{1}\bar{R}R_{P}}x_{1}+\frac{\left(R_{P}R_{T}+\bar{R}\right)}{C_{1}\bar{R}R_{P}}x_{2}+\frac{dP_{ext}}{dt}$$ (27)

$$\frac{dx_{2}}{dt}=\frac{\left(R_{P}R_{T}+\bar{R}\right)}{C_{2}\bar{R}R_{P}}x_{1}+\frac{-(R_{P}R_{T}+R_{P}R_{1}+\bar{R})}{C_{2}\bar{R}R_{P}}x_{2}+\frac{dP_{ext}}{dt}$$ (28)

The flow rates in terms of these new variables are:

$$q_{T}=\frac{-R_{2}x_{1}-R_{1}x_{2}}{\bar{R}}$$ (29)

$$q_{1}=\frac{-R_{2}x_{1}-R_{T}\left(x_{1}-x_{2}\right)}{\bar{R}}$$ (30)

$$q_{2}=\frac{R_{1}x_{2}-R_{T}\left(x_{1}-x_{2}\right)}{\bar{R}}$$ (31)

$$q_{P}=\frac{x_{1}-x_{2}}{R_{P}}$$ (32)

Eqs (27) and (28) can be written in matrix form as:

$$\frac{d\vec{X}}{dt}=A\vec{X}+\frac{dP_{ext}}{dt}\left[\begin{array}{c} 1\\ 1 \end{array}\right]$$ (33)

where

$$A=\left[\begin{array}{cc} \frac{-(R_{P}R_{T}+R_{P}R_{2}+\bar{R})}{C_{1}\bar{R}R_{P}} & \frac{\left(R_{P}R_{T}+\bar{R}\right)}{C_{1}\bar{R}R_{P}}\\ \frac{\left(R_{P}R_{T}+\bar{R}\right)}{C_{2}\bar{R}R_{P}} & \frac{-(R_{P}R_{T}+R_{P}R_{1}+\bar{R})}{C_{2}\bar{R}R_{P}} \end{array}\right]$$ (34)

and $\vec{X}={\left[x_{1}\;,\;\;x_{2}\right]}^{T}$.

If we consider the unforced system, where $\frac{dP_{ext}}{dt}=0$, Eq (33) becomes the autonomous, homogeneous, linear system $\frac{d\vec{X_{U}}}{dt}=A\vec{X_{U}}$, where $\vec{X_{U}}={\left[x_{1}\;,\;\;x_{2}\right]}^{T}$. The solution to this autonomous linear system is:

$$\vec{X_{U}}\left(t\right)=a_{1}e^{\lambda_{1}t}\vec{V_{1}}+a_{2}e^{\lambda_{2}t}\vec{V_{2}}$$ (35)

where *λ*_*i*_ are the eigenvalues of *A*, $\vec{V_{i}}$ are their corresponding eigenvectors, and *a*_*i*_ depend on the specific initial conditions.

To find explicit expressions for the eigenvalues and eigenvectors of the unforced system, we simplify our analysis by scaling time by $C_{1}\bar{R}R_{P}$. This transformation does not change the phase plane dynamics of the system. The matrix *A* (Eq (34)) becomes:

$$\hat{A}=\left[\begin{array}{cc} -(R_{2}R_{P}+\beta ) & \beta \\ \gamma \beta & -\gamma (R_{1}R_{P}+\beta ) \end{array}\right]$$ (36)

where $\beta =R_{T}R_{P}+\bar{R}$, and *γ* = *C*_1_/*C*_2_. The eigenvalues are then given by:

$$\lambda_{1}=\frac{Tr\left(\hat{A}\right)-\sqrt{Tr{\left(\hat{A}\right)}^{2}-4\text{Det}\left(\hat{A}\right)}}{2}$$ (37)

$$\lambda_{2}=\frac{Tr\left(\hat{A}\right)+\sqrt{Tr{\left(\hat{A}\right)}^{2}-4\text{Det}\left(\hat{A}\right)}}{2}$$ (38)

where:

$$Tr\left(\hat{A}\right)=-\left(\gamma R_{1}+R_{2}\right)R_{P}-\left(\gamma +1\right)\beta$$ (39)

$$\text{Det}\left(\hat{A}\right)=\gamma \left(R_{1}R_{2}R_{P}^{2}+\beta \left(R_{1}+R_{2}\right)R_{P}\right)$$ (40)

These eigenvalues will be real if $\Delta =\text{Tr}{\left(\hat{A}\right)}^{2}-4\text{Det}\left(\hat{A}\right)⩾0$.

In this system all resistance and compliance values must be positive. Using this simple physiological constraint we find that $Tr(\hat{A})<0$ and $\text{Det}(\hat{A})>0$ for all values of *C*_*i*_, *R*_*i*_ > 0. Additionally, it can be shown that $\Delta =\text{Tr}{\left(\hat{A}\right)}^{2}-4\text{Det}\left(\hat{A}\right)>0$, and thus the system has two real eigenvalues, *λ*_1_ < *λ*_2_ < 0, for all parameter values.

The corresponding eigenvectors are:

$$\vec{V_{1}}=\left[\begin{array}{c} 1\\ \frac{\lambda_{1}+R_{P}R_{2}+\beta }{\beta } \end{array}\right]\;\left(\text{fast}\right)$$ (41)

$$\vec{V_{2}}=\left[\begin{array}{c} 1\\ \frac{\lambda_{2}+R_{P}R_{2}+\beta }{\beta } \end{array}\right]\;\left(\text{slow}\right)$$ (42)

All solutions (given by Eq (35)) will tend to the single steady-state (*x*_1_, *x*_2_) = (0, 0) as *t* → ∞ by initially following the fast eigenvector $\vec{V_{1}}$ and then approaching the steady-state (more slowly) following the slow eigenvector $\vec{V_{2}}$. This behaviour is characteristic of solutions to linear ordinary differential equations.

In our model, we are looking for solutions where *q*_*P*_ ≥ 0. From Eq (32) we know that *q*_*P*_ = 0 on the line *x*_2_ = *x*_1_ and solutions above the line *x*_2_ = *x*_1_ will have *q*_*P*_ < 0 while solutions below the line *x*_2_ = *x*_1_ will have *q*_*P*_ > 0. Thus, to maintain positive unidirectional flow (*q*_*P*_ ≥ 0), solutions must lie on or below the line *x*_2_ = *x*_1_. Putting this information together with the knowledge that the unforced system will return to steady state (*x*_1_, *x*_2_) = (0, 0) following the slow eigenvector $\vec{V_{2}}$, we can conclude that solutions will be pushed into the region *q*_*P*_ < 0 if the slow eigenvector, $\vec{V_{2}}$ lies above the line *x*_2_ = *x*_1_. Consequently, in order to maintain unidirectional flow we must find conditions that ensure that the slow eigenvector lies on or below the line *x*_2_ = *x*_1_.

From Eq (42), $\vec{V_{2}}={\left[1,1\right]}^{T}$ when $\frac{\lambda_{2}+R_{P}R_{2}+\beta }{\beta }=1$. Rearranging this equation, we find that the slow eigenvector will lie along the line *x*_2_ = *x*_1_ when *γR*_1_ = *R*_2_. When perturbed by breathing, *P*_*ext*_ is applied along the vector [1, 1]^*T*^ (see Eq (33)). So in the case where *γR*_1_ = *R*_2_ and $\vec{V_{2}}={\left[1,1\right]}^{T}$, the system will oscillate (due to the forcing from breathing) along the line *x*_2_ = *x*_1_ and *q*_*P*_ = 0. This forms a useful boundary case. During *inspiration* the slow eigenvector will lie below the line *x*_1_ = *x*_2_ (*q*_*P*_ > 0) if $\frac{\lambda_{2}+R_{P}R_{2}+\beta }{\beta }>1$, which occurs when *γR*_1_ < *R*_2_. During *expiration*, the slow eigenvector will lie below the line *x*_1_ = *x*_2_ (*q*_*P*_ > 0) if $\frac{\lambda_{2}+R_{P}R_{2}+\beta }{\beta }<1$, which occurs when *γR*_1_ > *R*_2_. If both these conditions are satisfied, then the dynamics of the unforced system tells us that solutions will move quickly into the region *q*_*P*_ > 0 and will stay there. When the system is perturbed by breathing, the forcing *P*_*ext*_ is applied along the vector [1, 1]^*T*^, and thus will not move the solutions into the region *q*_*P*_ < 0.

This analysis gives the following conditions for unidirectional flow: *γR*_1_ ≤ *R*_2_ during inspiration and *γR*_1_ ≥ *R*_2_ during expiration.

#### Special case of even compliances

In the special case *γ* = 1 (that is, *C*_1_ = *C*_2_), the eigenvalues reduce to:

$$\lambda_{i}=-\frac{1}{2}\left[\begin{array}{c} \left(R_{1}+R_{2}\right)R_{P}+2\beta \pm \sqrt{{\left(R_{2}-R_{1}\right)}^{2}R_{P}^{2}+4\beta^{2}} \end{array}\right]$$ (43)

and the corresponding eigenvectors are:

$$\vec{V_{1}}=\left[\begin{array}{c} 1\\ \frac{\frac{1}{2}\left(R_{2}-R_{1}\right)R_{P}-\frac{1}{2}\sqrt{{\left(R_{2}-R_{1}\right)}^{2}R_{P}^{2}+4\beta^{2}}}{\beta } \end{array}\right]$$ (44)

$$\vec{V_{2}}=\left[\begin{array}{c} 1\\ \frac{\frac{1}{2}\left(R_{2}-R_{1}\right)R_{P}+\frac{1}{2}\sqrt{{\left(R_{2}-R_{1}\right)}^{2}R_{P}^{2}+4\beta^{2}}}{\beta } \end{array}\right]$$ (45)

If we set *R*_1_ = *R*_2_, the eigenvalues simplify further to:

$$\left[\begin{array}{c} \lambda_{1}\\ \lambda_{2} \end{array}\right]=\left[\begin{array}{c} -\frac{1}{2}\left(R_{1}+R_{2}\right)R_{P}-2\beta \\ -\frac{1}{2}\left(R_{1}+R_{2}\right)R_{P} \end{array}\right]$$ (46)

and the eigenvectors are:

$$\vec{V_{1}}=\left[\begin{array}{c} 1\\ -1 \end{array}\right]$$ (47)

$$\vec{V_{2}}=\left[\begin{array}{c} 1\\ 1 \end{array}\right]$$ (48)

When perturbed by breathing, this system will return along the line *x*_1_ = *x*_2_, where *q*_*P*_ = 0, which forms the boundary (zero flow) case. If *R*_2_ > *R*_1_, the slow eigenvector $\vec{V_{2}}$ (Eq (45)) lies below the line *x*_1_ = *x*_2_ during *inspiration* and *q*_*P*_ > 0. If *R*_1_ > *R*_2_, $\vec{V_{2}}$ (Eq (45)) lies below the line *x*_1_ = *x*_2_ during *expiration* and once again *q*_*P*_ > 0. Fig 4 shows a representative phase plane for *γ* = 1.

### Incorporating aerodynamic valving

The conditions for unidirectional flow stated above can only be satisfied if the resistances or compliances change between inspiration and expiration. As there is no evidence that the compliances would change during a single breathing cycle, we look instead at changing the resistances. Experimental findings have shown that the complicated anatomical structure causes effective aerodynamic valving, where the flow through the different pathways differs between inspiration and expiration. To reproduce this effect in our model we make the resistances *R*_1_ and *R*_2_ dependent on flow direction.

Inspiratory valving is incorporated into our model by increasing *R*_2_ when *q*_2_ < 0 (see Fig 2) to reduce the negative *q*_2_ flow during inspiration. The resistance *R*_2_ is:

$$R_{2}=R_{2,exp}+(R_{2,insp}-R_{2,exp})H\left(P_{J}-P_{2}\right)$$

where **H** denotes the Heaviside function, *R*_2,*exp*_ is the physiological value for resistance to flow in the preferred direction (*q*_2_ > 0) and *R*_2,*insp*_ is the higher effective resistance value during inspiration due to the inspiratory valving.

Expiratory valving is incorporated into our model by increasing *R*_1_ when *q*_1_ < 0 to prevent flow from the caudal airsacs during expiration (see Fig 2). The resistance *R*_1_ is:

$$R_{1}=R_{1,insp}+(R_{1,exp}-R_{1,insp})H\left(P_{1}-P_{J}\right)$$

where **H** denotes the Heaviside function, *R*_1,*insp*_ is the physiological value for resistance to flow in the preferred direction (*q*_1_ > 0) and *R*_1,*exp*_ is the higher effective resistance value during inspiration due to the expiratory valving.

From Eq (30) the flow *q*_1_ = 0 when $x_{2}=\frac{R_{2}+R_{T}}{R_{T}}x_{1}$, for *q*_*P*_ > 0 this line lies in the lower left quadrant of the (*x*_1_, *x*_2_)-phase plane. From Eq (31) the flow *q*_2_ = 0 when $x_{2}=\frac{R_{T}}{R_{1}+R_{T}}x_{1}$, for *q*_*P*_ > 0 this line lies in the upper right quadrant of the (*x*_1_, *x*_2_)-phase plane. From Eq (29) we can show that inspiration (*q*_*T*_ > 0) occurs in the region $x_{2}<-\frac{R_{2}}{R_{1}}x_{1}$, and expiration (*q*_*T*_ < 0) occurs in the region $x_{2}>-\frac{R_{2}}{R_{1}}x_{1}$.

**Fig 13:**
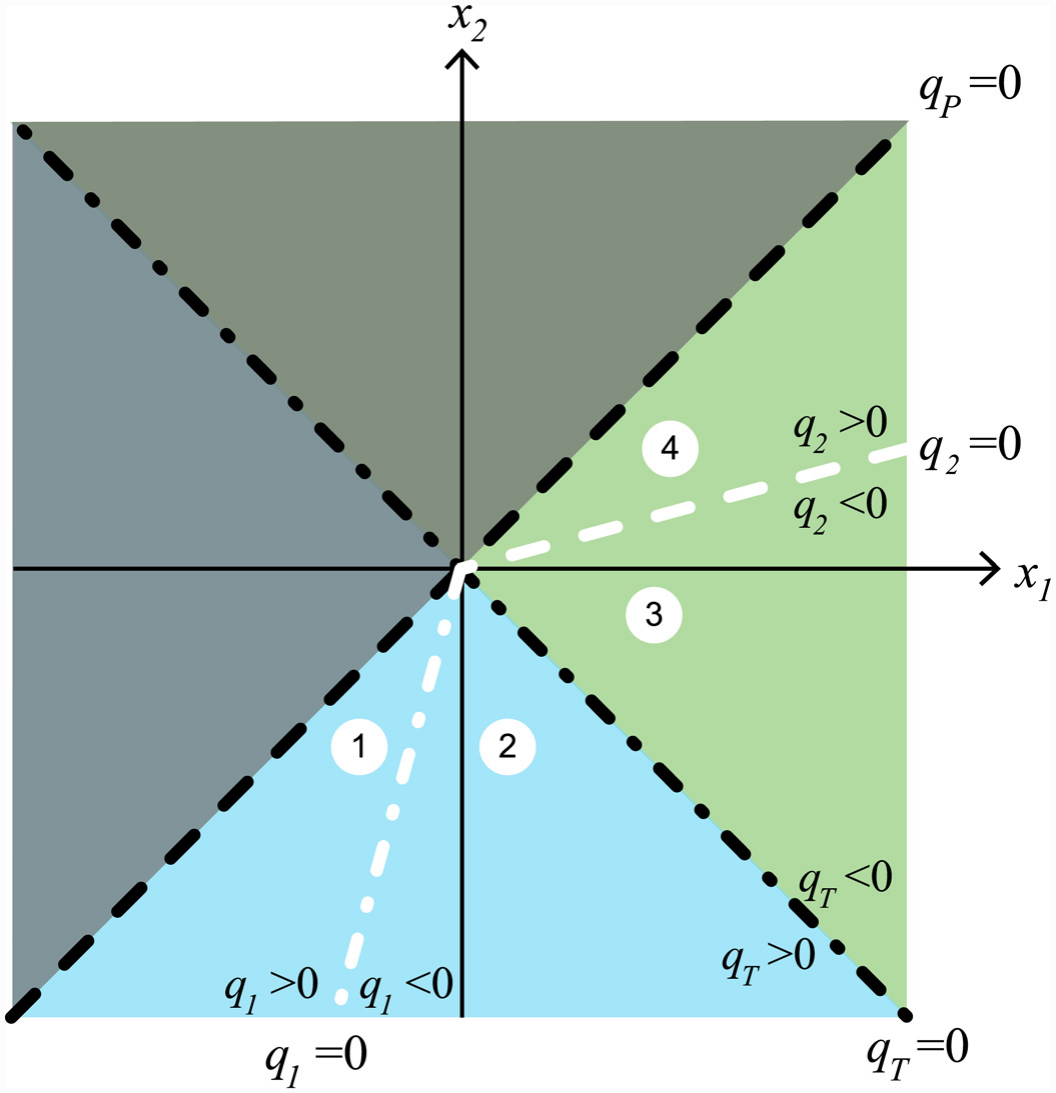
The position of zero flow points *q*_*T*_ = 0, *q*_1_ = 0, and *q*_2_ = 0 shown in the phase plane. The grey shaded region above the long dashed line *x*_2_ = *x*_1_ is where *q*_*P*_ < 0, and the unshaded region below the line *x*_2_ = *x*_1_ is where *q*_*P*_ > 0. The blue shaded region indicates where *q*_*T*_ > 0, and the green shaded region where *q*_*T*_ < 0. The value of *R*_1_ changes on the line *q*_1_ = 0 such that: *R*_1_ = *R*_1,*insp*_ in region (1), and *R*_1_ = *R*_1,*exp*_ in regions (2), (3), and (4). The value of *R*_2_ changes on the line *q*_2_ = 0 such that: *R*_2_ = *R*_2,*exp*_ in region (4), and *R*_2_ = *R*_2,*insp*_ in regions (1), (2), and (3).

Combining all this information we can sketch the flow direction transitions onto the phase plane (Fig 13), where:

- In region (1): *q*_*T*_ > 0, *q*_1_ > 0, and *q*_2_ < 0, so *R*_1_ = *R*_1,*insp*_, *R*_2_ = *R*_2,*insp*_.
- In region (2): *q*_*T*_ > 0, *q*_1_ < 0, and *q*_2_ < 0, so *R*_1_ = *R*_1,*exp*_, *R*_2_ = *R*_2,*insp*_.
- In region (3): *q*_*T*_ < 0, *q*_1_ < 0, and *q*_2_ < 0, so *R*_1_ = *R*_1,*exp*_, *R*_2_ = *R*_2,*insp*_.
- In region (4): *q*_*T*_ < 0, *q*_1_ < 0, and *q*_2_ > 0, so *R*_1_ = *R*_1,*exp*_, *R*_2_ = *R*_2,*exp*_.

Note that the *q*_*T*_ = 0 transition always occurs in the lower right quadrant, where *q*_1_ < 0 and *q*_2_ < 0. This means that we can define inspiration as the region $x_{2}<-\frac{R_{2,insp}}{R_{1,exp}}x_{1}$, and expiration as the region $x_{2}>-\frac{R_{2,insp}}{R_{1,exp}}x_{1}$. Also note that the transition through regions (2) and (3) is very fast.

### Implementation

We used Matlab’s event detection in conjunction with the solver ode23 to change *R*_1_ and *R*_2_ values when *q*_1_ and *q*_2_ crossed through zero. Starting at region (1) (*q*_1_ > 0, *q*_2_ < 0) the sequence for one breath is:

- set *R*_1_ = *R*_1,*insp*_, and *R*_2_ = *R*_2,*insp*_, and solve until *q*_1_ = 0 (region 1),
- set *R*_1_ = *R*_1,*exp*_, leave *R*_2_ = *R*_2,*insp*_, and solve until *q*_2_ = 0 (regions 2 & 3),
- leave *R*_1_ = *R*_1,*exp*_, set *R*_2_ = *R*_2,*exp*_, and solve until *q*_2_ = 0 (region 4),
- leave *R*_1_ = *R*_1,*exp*_, set *R*_2_ = *R*_2,*insp*_, and solve until *q*_1_ = 0 (regions 2 & 3).

This sequence was then repeated for as many breaths as required until steady state was reached. Steady state was numerically defined as being reached when the area under the curve *q*_*T*_ was zero (less than 1 × 10^−5^) over a single breath. That is, the flow into the bird was equal to the flow out of the bird in each breath.

The volumetric flow (area under the *q*_*i*_ curves) through each segment was found numerically using the trapezoidal method.

In the results presented, a step size of *δt* = 1 × 10^−4^ was used. The step size was reduced in several cases to test convergence and the results were consistent.

Note: We state and discuss resistances with the units cmH_2_O/L⋅s, but use the units mL/cmH_2_O for compliances and mL for volumes, based on common practice in the field. When implementing the model it is important to use consistent units (mL or L only).

### Selecting model parameters

We selected parameters to match duck respiratory systems, as we have the best data on airsac compliance and ventilation for this species. The default parameter values given in Table 1 are used for all the numerical calculations unless otherwise is indicated in the figure legends or in the text. Below we explain in more detail how we chose the specific parameters.

#### Resistances

*R*_2,*exp*_ = 5 cmH_2_O/L⋅s and *R*_*P*_ = 2.5 cmH_2_O/L⋅s are chosen to match physiological values measured in ducks [42]. There are no direct measurements of *R*_1,*insp*_, but we select *R*_1,*insp*_ = 1 cmH_2_O/L⋅s to match measurements of the overall lower respiratory system resistance in a single side (*R*_1,*insp*_ + *R*_*P*_ + *R*_2,*exp*_ ≈ 9 cmH_2_O/L⋅s) [42, 43] and measurements that show that the pressure drop between the primary bronchi and the caudal airsacs is very small [34]. The tracheal resistance *R*_*trachea*_ = 1 cmH_2_O/L⋅s is chosen to match the resistance obtained from measurements in [43] and checked against the resistance to laminar flow in a rigid tube calculated for the approximate length and diameter [1, 2, 44, 45]. The resistance across the constriction known as the segmentum accelerans *R*_*EPPB*_ = 8 cmH_2_O/L⋅s is chosen to generate observed pressure drop [34]. The overall respiratory system resistance can be calculated as *R*_*total*_ = 2*R*_*trachea*_ + *R*_*EPPB*_ + (*R*_1,*insp*_ + *R*_*P*_ + *R*_2,*exp*_)/2. For our chosen parameter values *R*_*total*_ = 14.25 cmH_2_O/L⋅s, which is consistent with measurements [42, 43, 46, 47].

#### Valving

To implement valving, we chose *R*_2,*insp*_ = 100 × *R*_1,*insp*_ to produce inspiratory valving efficacy in physiological range ≈96 − 100% [14, 33, 34], and chose *R*_1,*exp*_ = 10 × *R*_2,*exp*_ to produce expiratory valving efficacy in physiological range ≈88% for ducks at rest [14, 48]. We note here that measurements of expiratory valving efficacy are often calculated using the amount of air that flows through the lungs *q*_*P*_ as a proportion of the total amount of air that flows out of the caudal airsacs during expiration:

$$\text{Expiratory}\;\;\text{valving}\;\;\text{efficacy}=\frac{\int_{\text{EXP}}{\text{q}}_{\text{P}}\;\text{dt}}{\int_{\text{EXP}}{\text{q}}_{\text{P}}+\left(-{\text{q}}_{1}\right)\;\text{dt}}$$ (49)

This differs slightly from our definition of expiratory valving efficacy (see Eq (4)), and will give a lower estimate of the valving efficacy compared to our definition. However, using the above definition, our chosen parameters give 81% expiratory valving efficacy, which is still in the experimentally measured range of 76–95%.

#### Total airsac compliance

Because of experimental limitations, the compliance of the airsacs has not been measured; measurements have only been made of the total system, including the body wall. One technique that is used for measuring the compliance is to change the external pressure in a box around an anaesthetized bird and measure the resulting volume flow into or out of the animal. The slope *ΔV*/*ΔP* then gives a measure of the ‘static compliance’ of the total system. Experiments in chickens and ducks estimate the compliance at between 10–30 mL/cmH_2_O [43, 47]. A different technique for measuring compliance is to apply small oscillations in volume (at frequencies much higher than resting breathing frequencies) to spontaneously breathing birds, and fitting the response to a series R-I-C (resistance-inertance-compliance) model. Using this technique the ‘dynamic compliance’ of ducks is estimated at 7.7 mL/cmH_2_O [43]. However, this technique is very dependent on the chosen model being fitted, and relies on the overall resistance being constant throughout the breathing cycle. The differences in compliance measured with different techniques suggests that the overall compliance of the system is sensitive to body position and muscular tone.

**Fig 14:**
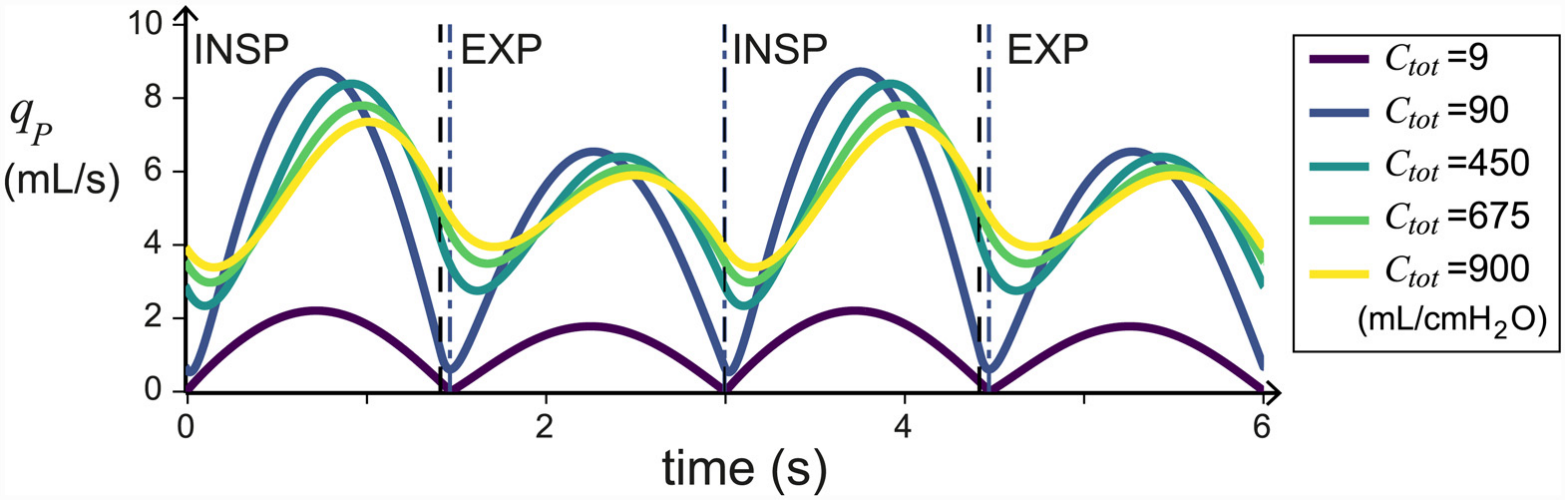
The shape of the flow *q*_*P*_ smoothes out as *C*_*tot*_ increases. This figure plots the flow through the parabronchi *q*_*P*_ against time for a range of *C*_*tot*_, with all traces aligned such that the beginning of inspiration is at *t* = 0. The parameter *γ* = 1, other parameters are given in Table 1. Note: the transition from inspiration to expiration happens at close to the same time for *C*_*tot*_ ≥ 90 mL/cmH_2_O and is shown as a black dashed line. The time of the transition for *C*_*tot*_ = 9 mL/cmH_2_O is shown with a dark blue dot-dashed line.

The elastance of the airsacs is found to be approximately 1/20th of the overall elastance [49], this means that we would expect the compliance of the airsacs to be approximately 20 times higher than the compliance of the overall system (recall that compliance = 1/elastance). Because of this uncertainty in the compliances, Urushikubo *et al*.[30] use compliances ranging from 0.009 to 900 mL/cmH_2_O for their lumped parameters model. In this work, we find that varying the total compliance does not affect many of the qualitative dynamics of the flow. However, with low compliances (less than 100 mL/cmH_2_O), the flow through the parabronchi is sharply changing between inspiration and expiration, as shown in Fig 14. We find that compliances over 100 mL/cmH_2_O produce airflow dynamics that vary smoothly and match experimental observations [10] much better.

#### Ratio of airsac compliances

**Fig 15:**
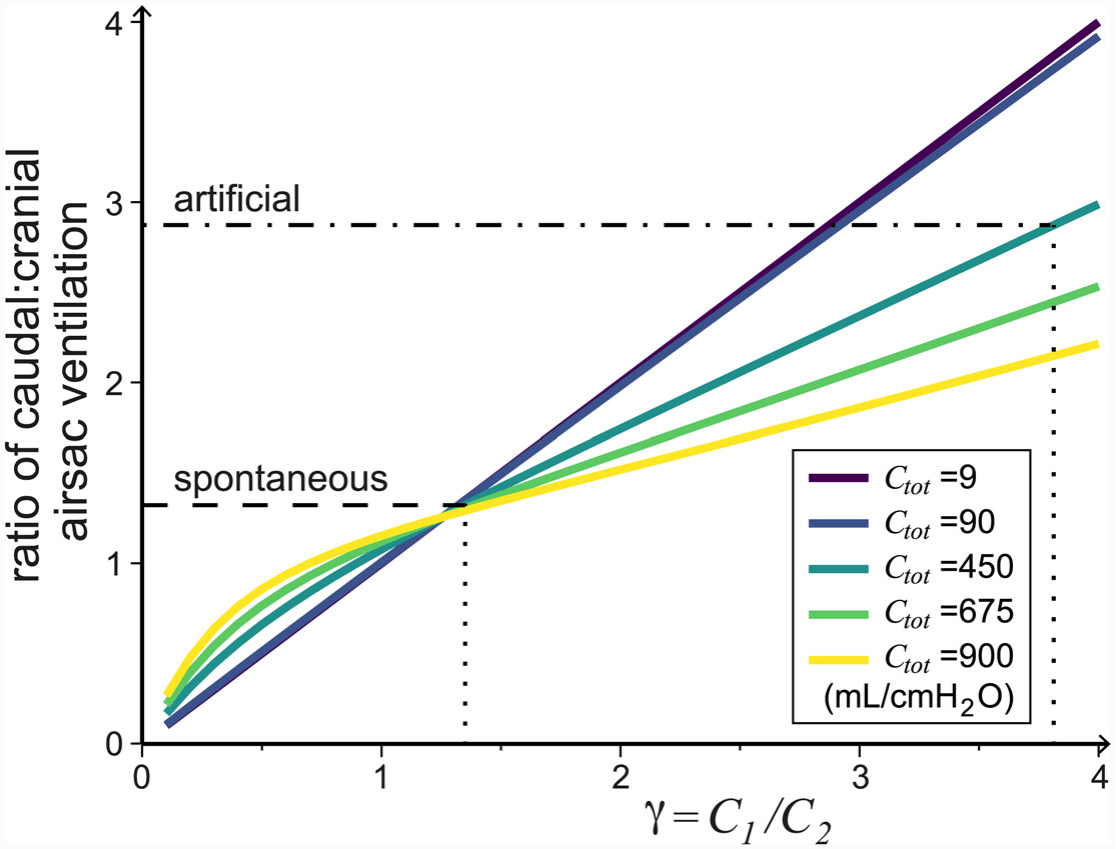
The ratio of caudal to cranial airsac ventilation increases as the ratio of airsac compliances *γ* = *C*_1_/*C*_2_ increases, i.e. the volume of the caudal airsacs changes more than that of the cranial airsacs. The experimentally measured ratio [38] in spontaneous breathing (dashed line) and artificial ventilation (dot-dashed line) ducks are shown. For different *C*_*tot*_ values, we would require slightly different *γ* values to match the experimental findings. The required *γ* values in each case for *C*_*tot*_ = 450 mL/cmH_2_O are shown with vertical dotted lines. All other parameters can be found in Table 1.

To determine the ratio of airsac compliances, *γ*, we looked at the phase of airflow through the parabronchi [10, 11] and used data on the ventilation ratios of the cranial and caudal sets of airsacs [13, 38, 47]. Experiments found that for spontaneously breathing ducks the effective ventilation of the caudal airsacs is 56.9% of the total ventilation of all airsacs [38]. In our model, we can achieve this ventilation ratio for different *γ* values depending on *C*_*tot*_, e.g. if we choose *C*_*tot*_ = 450 mL/cmH_2_O, we would need *γ* = 1.35 (the dashed line in Fig 15). However, for all compliances we found that *γ* > 1 (i.e., *C*_1_ > *C*_2_) for spontaneous breathing in ducks. Additionally, in relaxed (anaesthetized) conditions the ventilation ratio is 74.2% to the caudal airsacs, which is thought to be due to large increases in the compliance of the caudal airsacs [38]. To achieve this with *C*_*tot*_ = 450 mL/cmH_2_O we would require *γ* = 3.81 (the dot-dash line in Fig 15), where *C*_1_ > *C*_2_ as would be expected.

#### Forcing

In all our simulations we used the sinusoidal function given in Eq (11) to model the pressure *P*_*ext*_ outside the airsacs. The amplitude of the change in pressure due to breathing, *P*_*amp*_ = 0.5 cmH_2_O, is chosen to match the tidal volumes [38, 50] and pressures [15] seen experimentally. The period *T* = 3s is chosen to match the period of respiration of ducks at rest [9, 13, 38, 50]. Using the same *P*_*ext*_(*t*) for both sets of airsacs is justified, as we can consider the thoracic-abdominal cavity (coelom) which contains all the airsacs as a single compartment with uniform pressures [51]. Experiments show that the baseline pressure in the thoracic-abdominal cavity (coelom), *P*_*c*_, can vary depending on physiological conditions, i.e. when crowing roosters exhibit a transient increase in *P*_*c*_ up to 40 cmH_2_O above atmospheric pressure [52]. In this paper we choose *P*_*c*_ = *P*_*atm*_ for spontaneous breathing. This choice will not affect the airflow dynamics, but will alter the calculation of volumes.

#### Airsac volumes

The total volume of the cranial airsacs is found to be less than that of the caudal airsacs in a lot of avian species [2, 53]. In ducks, the peak (end-inspiration) volumes of the caudal and cranial airsacs during spontaneous breathing were found to be 235.4mL and 221.9mL, respectively [38]. We choose *V*_1,*res*_ = 105.6mL and *V*_2,*res*_ = 103.6mL for a single side as this gives the experimental peak volumes. In this paper, with *P*_*c*_ = *P*_*atm*_, the steady state volumes in the absence of changing pressures due to breathing $\left(\frac{dP_{ext}}{dt}=0\right)$ are *V*_1_ = *V*_1,*res*_ and *V*_2_ = *V*_2,*res*_. This can be calculated from Eqs (9) and (10) with *P*_1_ = *P*_2_ = *P*_*atm*_ and *P*_*amp*_ = 0.

## Supporting Information

S1 Fig Varying the compliance ratio or the total compliance does not change the overall efficiency substantially. **A:** shows the effect of varying the compliance ratio, *γ* = *C*_1_/*C*_2_, for five different *C*_*tot*_ values. **B:** shows the effect of varying the total compliance, *C*_*tot*_, for five different *γ* values.
(EPS)

S2 Fig Varying the resistance ratio or the total compliance only has a small effect on the I:E parabronchial volume flow ratio. **A:** shows the effect of varying the resistance ratio, *R*_1,*insp*_/*R*_2,*exp*_, while keeping the total resistance constant (*R*_1,*insp*_ + *R*_2,*exp*_ = 6 cmH_2_O/L⋅s), for five different *γ* values. **B:** shows the effect of varying the total compliance, *C*_*tot*_, for five different *γ* values.
(EPS)

S3 Fig Varying the compliance ratio or the total compliance does not affect the I:E time ratio substantially. **A:** shows the effect of varying the compliance ratio, *γ* = *C*_1_/*C*_2_, for five different *C*_*tot*_ values. **B:** shows the effect of varying the total compliance, *C*_*tot*_, for five different *γ* values.
(EPS)
